# Rapid phenotypic evolution with shallow genomic differentiation during early stages of high elevation adaptation in Eurasian Tree Sparrows

**DOI:** 10.1093/nsr/nwz138

**Published:** 2019-09-12

**Authors:** Yanhua Qu, Chunhai Chen, Ying Xiong, Huishang She, Yong E Zhang, Yalin Cheng, Shane DuBay, Dongming Li, Per G P Ericson, Yan Hao, Hongyuan Wang, Hongfeng Zhao, Gang Song, Hailin Zhang, Ting Yang, Chi Zhang, Liping Liang, Tianyu Wu, Jinyang Zhao, Qiang Gao, Weiwei Zhai, Fumin Lei

**Affiliations:** 1 Key Laboratory of Zoological Systematics and Evolution, Institute of Zoology, Chinese Academy of Sciences, Beijing 100101, China; 2 BGI Genomics, BGI-Shenzhen, Shenzhen 518084, China; 3 University of Chinese Academy of Sciences, Beijing 100049, China; 4 Center for Excellence in Animal Evolution and Genetics, Chinese Academy of Sciences, Kunming 650223, China; 5 Committee on Evolutionary Biology, University of Chicago, Chicago, IL 60637, USA; 6 Life Sciences Section, Integrative Research Center, Field Museum of Natural History, Chicago, IL 60605, USA; 7 Key Laboratory of Animal Physiology, Biochemistry and Molecular Biology of Hebei Province, College of Life Sciences, Hebei Normal University, Shijiazhuang 050024, China; 8 Department of Bioinformatics and Genetics, Swedish Museum of Natural History, SE-104 05 Stockholm, Sweden; 9 College of Life Sciences, Shaanxi Normal University, Xi'an 710119, China; 10 China National GeneBank, BGI-Shenzhen, Shenzhen 518120, China; 11 Human Genetics, Genome Institute of Singapore, Agency for Science, Technology, and Research, Singapore 138672, Singapore

**Keywords:** *de novo* genome, early stage adaptation, high elevation, muscle phenotypes, polygenic adaptation, regulatory evolution

## Abstract

Known as the ‘third polar region’, the Qinghai-Tibet Plateau represents one of the harshest highland environments in the world and yet a number of organisms thrive there. Previous studies of birds, animals and humans have focused on well-differentiated populations in later stages of phenotypic divergence. The adaptive processes during the initial phase of highland adaptation remain poorly understood. We studied a human commensal, the Eurasian Tree Sparrow, which has followed human agriculture to the Qinghai-Tibet Plateau. Despite strong phenotypic differentiation at multiple levels, in particular in muscle-related phenotypes, highland and lowland populations show shallow genomic divergence and the colonization event occurred within the past few thousand years. In a one-month acclimation experiment investigating phenotypic plasticity, we exposed adult lowland tree sparrows to a hypoxic environment and did not observe muscle changes. Through population genetic analyses, we identified a signature of polygenic adaptation, whereby shifts in allele frequencies are spread across multiple loci, many of which are associated with muscle-related processes. Our results reveal a case of positive selection in which polygenic adaptation appears to drive rapid phenotypic evolution, shedding light on early stages of adaptive evolution to a novel environment.

## INTRODUCTION

Organisms living at high elevations are exposed to cold temperatures and low levels of oxygen, imposing severe physiological constraints and challenges [1–3]. In birds and mammals, hypoxia is one of the most obvious selective pressures that drive physiological adaptation [4,5]. For examples, increases in hemoglobin-oxygen affinity, muscle fiber number and capillarization have been observed in several species, e.g. Bar-headed goose, Andean waterfowl [6–8], passerine [9], Japanese quail [10] and deer mice [11]. The Qinghai-Tibet Plateau (QTP), often known as the ‘third polar region’ [12], has an average elevation of 4500 m above sea level (m.a.s.l.), representing one of the harshest highland environments in the world. In many animals living on the QTP, low temperatures and hypoxic conditions have driven drastic phenotypic adaptations that enhance hypoxia resistance, cold tolerance and metabolic capacity [13–15]. Recent studies have found that many of these adaptive phenotypic changes are associated with genomic changes. For example, selection in genes that impact hypoxia response, energy metabolism, oxygen transport and skeletal development have been found in the Tibetan pig, yak, antelope, ground tit and chicken [16–22].

The Eurasian tree sparrow (*Passer montanus*, hereafter referred to as tree sparrow) is a human commensal that is widely distributed across Eurasia [23,24]. It is closely linked to human settlement and cultivation [23] and is likely to have occupied the QTP following the introduction of human agriculture [25]. Unlike most QTP species studied so far that are either highland endemic or locally domesticated, the tree sparrow is predominately associated with farmland and agricultural areas, feeding on grains and seeds from cultivated cereals [23,24]. Although humans colonized the QTP over 15 000 years ago [26], the rise of farming above 2000 meters, especially of cold-tolerant barley, remains fairly recent (e.g. since 3600 years ago) [27,28]. Despite this short history of agriculture, the highland tree sparrow displays a number of adaptive phenotypes that are similar to other highland species, such as increased body size and muscle mass [28,29]. The mechanisms driving their rapid colonization and persistence on the QTP remain largely enigmatic.

To date, most studies of high elevation adaptation have focused on highland species that are well differentiated from lowland sister lineages, representing later stages of adaptive evolution [16–22]. As a recent colonizer of the QTP, studying the tree sparrow can empower us to unravel the adaptive dynamics during the early stage of high elevation adaptation, a process that remains largely unexplored outside of humans. In this work, we first assembled and generated a high-quality *de novo* genome of the tree sparrow. By resequencing and comparing individuals from the QTP to individuals from lowland areas, we found that the tree sparrow colonized the QTP within the past few thousand years in concert with the rise of barley agriculture on the QTP, which allowed for permanent high elevation settlements. Despite this short colonizing time, highland tree sparrows have quickly adapted to the harsh highland environment by increasing muscle fiber area and capillarization in the flight and cardiac muscles, two organs that show large phenotypic divergence between the highland and lowland populations [30,31]. Through population genomic analysis, we identified an evolutionary process of polygenic adaptation, in which gene frequency shifts across multiple loci can drive rapidly phenotypic evolution [32,33]. By analyzing transcriptomic data with the context of muscle phenotypes, we found that multiple genes in the RhoA GTPase/Rho kinase (RhoA/ROCK)-mediated acto-myosin filament are strongly correlated with muscle phenotypes. Our results reveal a case of high elevation adaptation in which frequency changes across multiple loci appear to drive rapid phenotypic divergence during an early stage of adaptive evolution to a novel environment.

## RESULTS

### Genome sequencing, assembly and annotation

To build a high-quality *de novo* reference genome, we sequenced a total of 177 gigabases (Gb) of data from a lowland tree sparrow collected from Beijing, China (Supplementary Table 1, see supplementary note 1 for a detailed description of genome assembly and annotation). After gap-filling and removal of adapter sequences, short reads were assembled into a genome of 1.05 Gb with an N50 scaffold length of 11.16 Mb and an N50 contig length of 750 Kb (Supplementary Fig. 1 and Supplementary Tables 1–3), reflecting a substantial increase in genome quality compared to published avian genomes, which typically have an N50 scaffold length of ∼10 Mb and an N50 contig size of ∼150 kb [19,20,34,35]. Using a combination of homology-based comparison, *de novo* gene prediction and RNA-seq, we annotated 16 925 high-confidence protein-coding genes (Supplementary Tables 4–8), 99.5% of which have homologs in protein databases (Supplementary Table 9). The number of genes is comparable to those gene sets in recently published avian genomes (e.g. 13 036 in the great tit, 15 932 in gray-coated hooded crows and 17 462 in Tibetan chicken [20,34–36]). Using Benchmarking Universal Single-Copy Orthologs version 2 (BUSCO, aves_odb9) [37], we estimated that the assembly contains almost complete (95.4%) eukaryote BUSCO orthologs (Supplementary Table 10–11). In addition, 94.8% EST assembled transcripts are mappable to the genome assembly, suggesting high coverage of the transcribed gene set (Supplementary Table 12). Taken together, these results indicate that our sequencing and assembly have generated a high-quality draft genome of the tree sparrow that can serve as a powerful foundation for subsequent population genomic and transcriptomic analyses.

**Figure 1.**
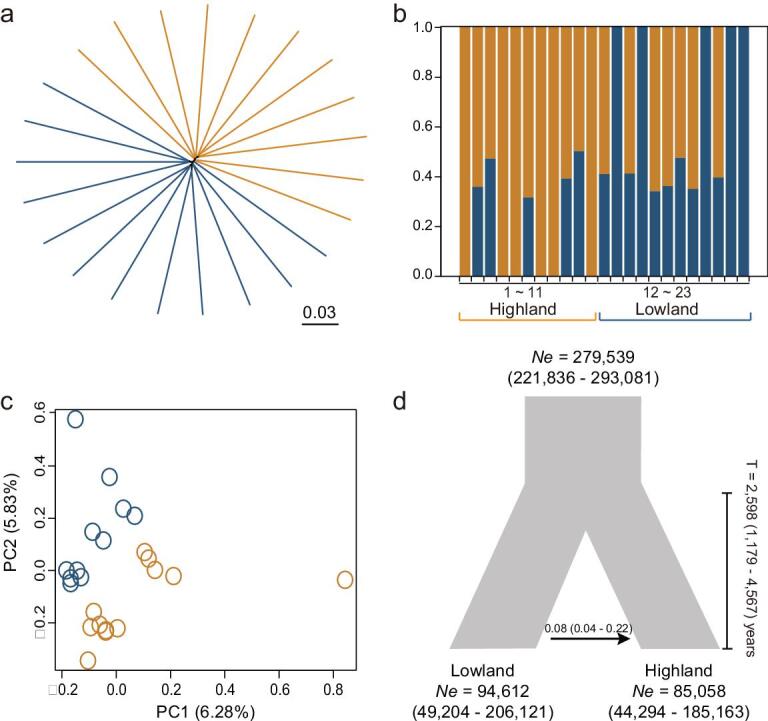
Population genetic structure of the Eurasian Tree Sparrow (*Passer montanus*). (a) Neighbor-joining tree based on genome-wide single-nucleotide polymorphisms (SNPs). (b) The genetic structure generated using FRAPPE [75]. The colors in each column represent the contribution from each subcluster. (c) Principal component analysis of SNPs from highland and lowland tree sparrows. The plot is based on the first two principal components. (d) Inferred demographic history of the highland and lowland tree sparrows.

### Shallow divergence between highland and lowland populations

To understand the evolutionary relationship and historical dynamics of the tree sparrow, we resequenced 11 highland birds sampled from Heimahe on the QTP (3200–3213 m.a.s.l., Supplementary Table 13) and 12 lowland birds from Beijing and Hebei (60–100 m.a.s.l., Supplementary Table 13). The mean sequence coverage was 17× per individual (range: 12–28×, Supplementary Table 14) and we identified 8.23 million single-nucleotide polymorphisms (SNPs). Nucleotide diversity (π), Tajima’s *D* values and linkage disequilibrium (LD) were similar between highland and lowland populations (nucleotide diversity, 2.27 e^−3^*vs.* 2.26 e^−3^; Tajima’s *D*, 1.194 *vs.* 1.264; LD mean *r*^2^, 0.108 *vs.* 0.103, Supplementary Table 15 and Supplementary Fig. S2). When calculating the level of population differentiation between the highland and lowland populations, the genome-wide mean *F*_ST_ was 0.026 (95% CI, 0–0.196) and *D*_XY_ was 4.5e^−4^ (95% CI, 0–0.0035), suggesting that the two populations are only weakly structured. We also confirmed this result from the neighbor-joining tree and population structure analysis (K = 1–5, with K = 1 getting the lowest cross-validation error, 0.63893, Supplementary Table 16) of all individuals Fig. 1a–b and Supplementary Fig. 3. When performing a principle component analysis (PCA) of SNP data, the two populations weakly differentiate along PC2 (5.8% of the total variation, Fig. 1c).

### Recent colonization of the Qinghai-Tibet Plateau

To infer the demographic history of the highland and lowland populations, we used FASTSIMCOAL 2.6 [38] to compare several different demographic histories, including a single population hypothesis (a constant and a changing population size model) and colonization hypotheses (three models with unidirectional and bi-directional gene flow between highland and lowland populations and three models allowing population sizes to change over time, Supplementary Fig. 3, Methods). Using Akaike information criterion (AIC), our data set supports a scenario of recent colonization with unidirectional gene flow from lowland to highland populations (Supplementary Table 17). When fitting this model to the two populations (using a mutation rate of 3.3e^−9^ [34] and a generation time of one year [23]), we estimated that the highland tree sparrow colonized the QTP approximately 2598 years ago (95% CI, 1179–4567 years ago) with an effective population size of 85 058 (95% CI, 44 294–185 163) (Fig. 1d). Estimates of gene flow were ∼0.08 from the lowland to highland populations (95% CI, 0.04–0.22) per generation (Supplementary Table 18). This inferred demographic history matches observations of a weak population differentiation from PCA and structure analyses, suggesting a recent colonization event for the highland tree sparrow.

### Rapid evolution in muscle phenotypes after colonizing the Qinghai-Tibet Plateau

In birds, muscle makes up the 25–35% of body mass, and birds generate endogenous heat primarily through muscle shivering [39,40]. Recent work has demonstrated how changes in muscle phenotypes (e.g. increases in muscle capillarity and oxidative muscle fibers) can improve metabolic capacity and enhance oxygen delivery in highland vertebrates [30,31,41,42]. These studies underscore the functional importance of muscle phenotypes in highland adaptation [30,31,41,42].

**Figure 2.**
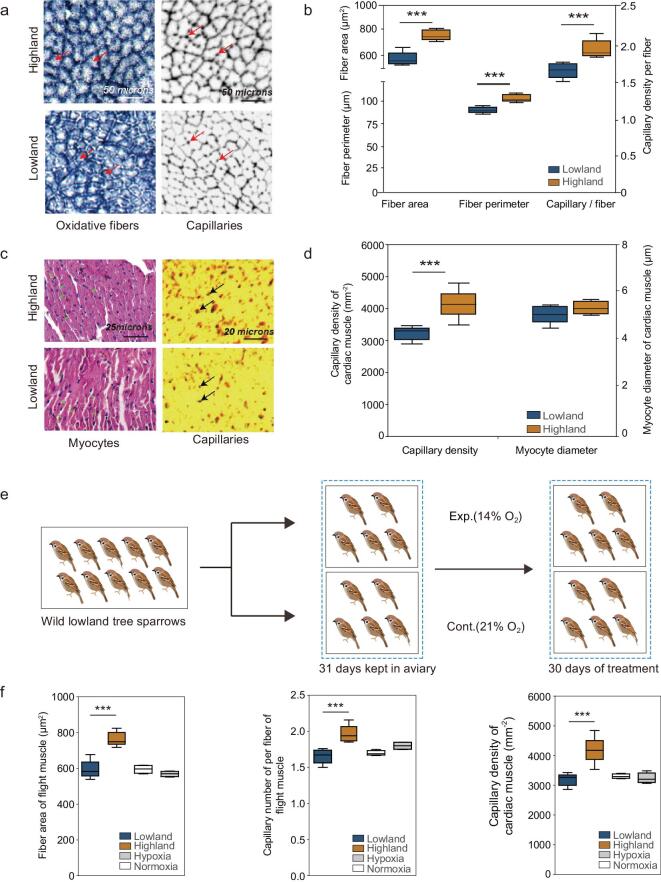
Histological analyses of the flight and cardiac muscle. (a) Representative images of the fast oxidative glycolytic fibers. Red arrows show fiber sizes (left) and capillaries (left) of the lowland (upper) and highland tree sparrows (lower), respectively. (b) The flight muscle in the highland tree sparrows has a significantly larger fiber area (*F*_1,11_ = 44.7, *P* < 0.001), perimeter (*F*_1,11_ = 47.29, *P* < 0.001) and capillary number per fiber than the lowland tree sparrows (*F*_1,11_ = 21.41, *P* < 0.001). Mean and standard error are shown. Blue boxes, lowland tree sparrows; orange boxes, highland tree sparrows. (c) Representative images of the cardiac muscle. Blue lines show myocyte diameters (right) and black arrows show capillaries (left) of the highland (upper) and lowland tree sparrows (lower), respectively. (d) The cardiac muscle in the highland tree sparrows has only slightly larger myocyte diameters (*F*_1,13_ = 2.99, *P* = 0.1) but significantly larger capillary density (*F*_1,13_ = 30.84, *P* < 0.001) than in the lowland tree sparrows. Mean and SE are shown. (e) Acclimation experiment protocol for the hypoxia-exposed lowland tree sparrows and control lowland tree sparrows. (f) The fiber areas of the flight muscle (left), the number of capillaries per flight muscle (middle) and the capillary density of the cardiac muscle (right) in the experimental birds (grey boxes) were similar to those of the control birds (white boxes) and the wild lowland birds (blue boxes), but all were reduced when compared to those of the highland tree sparrows (orange boxes).

To identify phenotypic changes in the muscle of highland tree sparrows, we measured muscle fibers and tissue vasculature (i.e. muscle capillarity) in the *pectoralis major* (the dominant flight muscle in birds, n = 12) and the cardiac muscle (n = 15), two organs that are critical to thermogenic capacity, oxygen transport and metabolism. We found that the average fiber size of fast oxidative (FO) fibers, the predominant muscle fiber type in the flight muscle of the small-bodied birds [43], was larger in the *pectoralis* of highland tree sparrows than in lowland tree sparrows, when measured as fiber area (759 *vs.* 582 μm^2^, *P* < 0.001) and as fiber perimeter (102 *vs.* 88 μm, *P* < 0.05, Fig. 2a and b, Supplementary Table 19). Consistent with the larger fiber size, we observed a higher capillary number in highland tree sparrows than in lowland tree sparrows (1.93 *vs.* 1.63 capillaries per muscle fiber, respectively; *P* < 0.001, Fig. 2a and b, Supplementary Table 19). Considering that muscle phenotypes could correlate with each other, we ran a linear regression of capillaries per fiber against FO fiber size. We found that fiber size significantly predicted capillaries per fiber (R^2^ = 0.6, *F*_1,11_ = 14.39, *P* < 0.01, Supplementary Fig. 4). This raises the question as to whether increased capillaries in highland tree sparrows is solely an effect of larger fiber area, or whether they have relatively more capillary once fiber size is accounted for. To explore this, we ran a general linear model setting the number of capillary per fiber as a dependent variable, highland/lowland as a fixed variable, and the fiber area as a covariant variable. The differences in the number of capillary per fiber between highland and lowland populations was no longer significant after controlling for fiber size (*F*_1,11_ = 2.68, *P* = 0.136), suggesting that the observed increase in the number of capillaries per fiber in highland birds is a consequence of larger fibers.

When we examined cardiac muscle, we found that the mean myocyte diameters of cardiac fibers were only slightly larger in the highland tree sparrows than in the lowland tree sparrows (5.25 *vs.* 4.95 μm, *P* = 0.1, Fig. 2c–d, Supplementary Table 20). In contrast to the slight increase in myocyte diameter, we observed a significant increase in capillaries in highland tree sparrows compared to lowland tree sparrows (4174 *vs.* 3201 capillaries per mm^2^, *P* < 0.001, Fig. 2c–d, Supplementary Table 20). A linear regression analysis confirmed that myocyte diameter of cardiac fiber did not predict capillary number (R^2^ = 0.09, *F*_1,9_ = 0.77, *P* = 0.41). A general linear model analysis showed that the number of capillaries also is significantly higher in highland tree sparrows than in lowland tree sparrows after controlling for the effect of the myocyte diameter (*F*_1,9_ = 48.23, *P* < 0.001). This result indicates that highland tree sparrows have relative increases in both cardiac capillary number and myocyte area.

**Figure 3.**
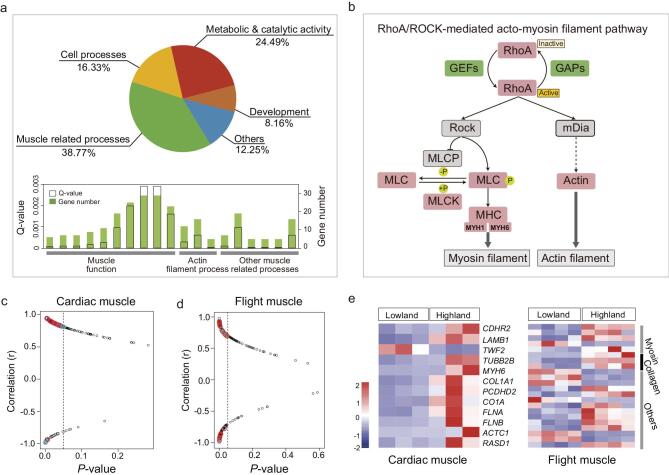
Multiple genes are associated with phenotypic variation in the muscle of highland tree sparrows. (a) Gene ontology (GO) analysis of the genes under divergent selection. The colors represent GO categories clustered into different functions, of which 39% of GO categories are related to muscle phenotypes (green). Gray bars, FDR value of significance; filled bars, number of genes in each GO. (b) Genes subject to divergent selection (green) and significantly transcriptional change (pink) in the highland tree sparrows are displayed in the RhoA/ROCK signaling pathway. (c-d) Correlations between expression profiles of the differentially transcribed genes with muscle phenotype variation. Blue and red together indicate genes related to all muscle processes while red specifies the genes in the RhoA/ROCK signaling pathway. (e) Plots of log-transformed ratio expression rates of genes in the RhoA/ROCK signaling pathway (red and blue indicate up-regulated and down-regulated genes in the highland tree sparrows).

### Phenotypic plasticity unaccounted for divergent muscle phenotypes in an acclimation experiment

Although we found significant phenotypic differences in flight and cardiac muscles between highland and lowland tree sparrows, the shallow genetic divergence suggests that processes other than genetic differentiation also may be at play. We hypothesized that the observed phenotypic change in highland population is a plastic, acclimation response to high elevation environments. To test this hypothesis, we simulated high elevation conditions using lowland tree sparrows (n = 5), experimentally exposing lowland individuals to an oxygen level equivalent of 3200 m.a.s.l, after which we analyzed muscle phenotypes to look for changes (Fig. 2e). Five lowland individuals were used as a control. We considered only oxygen level in this acclimation experiment, not temperature, because changes in muscle phenotypes have been observed in tree sparrows at high elevations, but not at high latitudes (low temperature areas). This dynamic suggests that low oxygen level may be the dominant environmental pressure at the high elevation region [4,5]. After one month of hypoxia exposure, we found that the number of capillaries per fiber, the area of FO fibers in the flight muscle and the capillary density of cardiac muscles in experimental birds were similar to those of the control birds and the natural lowland population (Fig. 2f). These results suggest that short-term acclimation might have little impact on the observed muscle phenotypes in the highland population.

### Polygenic adaptation and transcriptomic divergence in muscle phenotypic changes

Given that the acclimation response was negligible for muscle phenotypes, we wondered whether genetic changes in the form of frequency shifts across many loci (i.e. polygenic adaptation) might associate with the observed phenotypic differences. To identify loci under divergent selection, we calculated *F*_ST_ for all SNPs between highland and lowland tree sparrows. To nominate the cutoff values, we first generated genome-wide nominal values under the inferred demographic history, using a stringent cutoff to identify potential regions of selection (using top 1% quantile of the simulated *F*_ST_ distribution, i.e. *F*_ST_ > 0.24, Methods). Using a combination of *F*_ST_ together with the change in genetic diversity between the two populations (θπ_lowland/θπ_highland>1.06, Methods), we identified 87 genes from 361 putatively selected regions that were strongly differentiated between the two populations. Gene Ontology (GO) analysis of the 87 positively selected genes (PSGs) using KOBAS [44] identified 49 significantly GO terms, which are enriched for muscle related processes, metabolic and catalytic activity, cell processes and development (Fig. 3a). Of these, the largest proportion of the GO terms (around 39%) was found to be related to muscle processes (Fig. 3a and Supplementary Fig. 5). Moreover, of the 87 PSGs under divergent selection, 20 were related to muscle processes (Supplementary Table 21 and Supplementary Fig. 6). This proportion is significantly higher than the muscle-related genes in the genome background (630 muscle related genes out of 16 925 genes, χ^2^ test, *P* = 1.101e^−15^), suggesting that PSGs related to muscle processes are over-presented in the highland tree sparrow. To further confirm these results, we used an alternative approach, e.g. Sweepfinder, and we found that the top selected regions also are enriched for similar pathways for muscle-related functions (Supplementary Table S22).

Because a large proportion of the top selected regions do not contain any genes, we hypothesized that changes in gene regulation also may contribute to evolved differences in muscle phenotypes

[30,31,45]. We subsequently compared levels of gene expression in flight (n = 8) and cardiac muscles (n = 6) between the two populations (Methods, Supplementary Tables 23), and identified 204 (flight muscle) and 134 (cardiac muscle) genes that were differentially expressed between highland and lowland populations (Supplementary Tables 24–25). Although the PSGs do not overlap with the differentially expressed genes (DEGs) in the flight and cardiac muscles, we saw strong enrichment in similar GO terms related to muscle processes (Fig. 2 and Supplementary Fig. 7). We hypothesized that PSGs and DEGs overlapped at the pathway level (i.e. KEGG pathways). We found that ‘focal adhesion’ (KEGG 04510) and ‘tight junction’ (KEGG 04530) pathways are among the top enriched pathways for both PSGs and DEGs (Supplementary Table 26). Among these pathways, PSGs (such as *ARHGAPs*, *GEFs* and *CTNNA3)* and DEGs (such as *COL1As, MYLKs, FIGF, FLNA, MYH1* and *MYH6*) jointly regulate the actin and myosin filament polymerization, i.e. RhoA/ROCK signaling transduction (Fig. 3b, Supplementary Fig. 9 and Supplementary Table 24–25), an important process that regulates muscle gene transcripts and their downstream cytoskeletal remodeling [46,47]. RhoA regulatory function is controlled by the interaction between RhoGEFs and RhoGAPs [48–50]. We found that five genes (*OBSCN*, *TRIO*, *ARHGAP15*, *ARHGAP18* and *ARHGAP39*) that encode these regulatory proteins are under divergent selection, while genes encoding RhoA and its downstream effectors (e.g. MLCKs, MLC, MYHs and actin) are differentially expressed in the highland tree sparrow.

**Figure 4.**
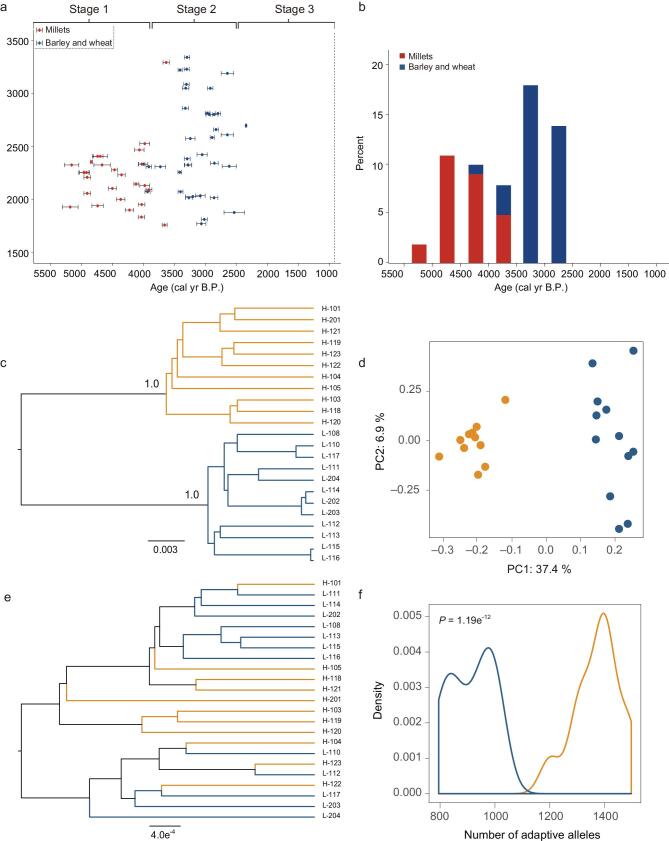
(a) Radiocarbon dates of charred grains (close dots with 2 error bars) from known archaeological sites on the Qinghai-Tibetan Plateau (QTP; modified from Fig. 2 and Table S1 in Chen *et al*. [26]). Red dots indicate millets and blue dots indicate barley and wheat. Stage 1, millet-based agriculture; stage 2, mixed agriculture; stage 3; barley-based agriculture. (b) Percentages of different crop remains from the investigated sites on the QTP from 5500 to 2400 years. Barley sites have increased after 3600 years ago. This figure is modified from Fig. S6 in Chen *et al*. [26]. (c) Single-nucleotide polymorphism (SNP) phylogeny generated with SNAPP using the highly divergent (HD)-SNPs (*F*_ST_ > 0.24) from 87 positively selected genes (PSGs). (d) Principal component analysis of same HD-SNPs as in panel d. (e) The phylogeny of SNP generated with SNAPP based on 956 randomly selected SNPs. (f) Number of adaptive alleles (i.e. allelic dosage) at the HD-SNPs from 87 PSGs (*t* test, *P* = 1.19e^−12^). (c-f) Highland population, orange; lowland population, blue.

To explore if transcriptional changes of genes in the RhoA/ROCK signaling transduction are strongly correlated with muscle phenotypes, we correlated levels of gene expression of the DEGs with muscle phenotypes of highland and lowland individuals using the first eigenvectors from PCA that summarize 90% and 82% variation of three flight and two cardiac muscle phenotypes, respectively. We ranked correlation coefficient and used a cutoff of *P* < 0.05 to select the genes with the strongest correlation with muscle phenotypes. Thirty-two and twenty-three genes related to muscle processes significantly correlated with flight and cardiac muscle phenotypes, respectively (Fig. 3c–d). Of these genes, a majority (20 genes, 63% in flight muscle; 12 genes, 52% in cardiac muscle) are part of the RhoA/ROCK signaling transduction (Fig. 3a and 3e), suggesting that this signaling pathway is important in regulating muscle phenotypes in the highland tree sparrow.

## Discussion

By combining phenotypic comparison with genomic and transcriptomic analyses, we uncovered a strong case of high elevation adaptation in which polygenic adaptation appears to drive rapid phenotypic divergence shortly after colonizing the QTP. Resequencing highland and lowland tree sparrows revealed a recent colonization event of the QTP with shallow population divergence. Phenotypic comparisons found that highland tree sparrows have changed their muscle phenotypes, which would have downstream consequences on oxygen delivery and oxidative metabolism. Genetic analyses of the two populations point to a process of polygenic adaption in highland tree sparrows from genomic data, whereby allele frequency shifts across multiple gene loci, driving phenotypic divergence between populations. Without synchronous selective sweeps, this polygenic process may mitigate some of the cost of selection associated with hard sweeps, allowing phenotypes to change over a relatively short period of time [51].

The estimated time of colonization of the QTP by the tree sparrow largely overlaps with the origin of agriculture on the QTP. The archeological record shows that Tibetans began cultivating millets as early as 5000 years ago (millet-based agriculture, Fig. 4a–b). The short growing season, low labor input and high frost-sensitivity of millets largely restricted cultivation to lower elevations (<2500 m.a.s.l.) and Tibetans lived mainly as hunters and gatherers with a low dietary input from millet [26,27]. Agriculture at higher elevations, however, became possible as cold-tolerant wheat and barley replaced millets, eventually becoming the dominant crop on the QTP between 3500 to 2500 years ago (barley-based agriculture, Fig. 4a–b, see also Chen *et al*. [26]). These high-yield crops enabled the establishment of permanent human settlements at high elevation on the QTP, creating an available niche for the tree sparrows (see also other human commensal house sparrows, *Passer domesticus* [52]).

The study of the tree sparrow also provides a unique opportunity to disentangle the sequential evolution of multiple phenotypes that contribute to high elevation adaptation. Even with shallow genetic divergence, muscle related phenotypes have rapidly evolved, likely facilitating early stages of high elevation adaptation. Unlike other highland birds where muscle adaptation is driven by increases in oxidative fiber number in the flight muscle [30,31,53], highland tree sparrows show increases in fiber area. However, this strategy is not optimal because a larger fiber area increases the distance oxygen travelling through the cells, reducing the capacity for oxygen diffusion [41,54,55]. Moreover, our acclimation experiment has several limitations (e.g. only one-month hypoxia acclimation and only measuring adult muscle response without taking into account developmental plasticity), and we cannot completely rule out the possibility of a suite of other factors affecting the muscle phenotypic plasticity (e.g. muscle developmental plasticity, epigenetic regulation, low temperature and other region-specific factors associated with QTP, a detailed discussion on limitations of the current study are presented in the supplementary note 2). Recent acclimation experiments in vertebrates vary in time from a few weeks [56–59] to several months [60,61]. Our acclimation may be on the short side, especially when considering a phenotypic trait such as muscle mass, which may acclimate slowly. We expect that acclimation of other adaptive traits, e.g. hematocrit concentration (supplementary note 2) and transcriptional changes, might be accounted for within a one-month period [62]. Nevertheless, even with these limitations, this study provides a unique opportunity to look into the early stage of high elevation adaptation where polygenic adaptation appears to drive adaptive phenotypes to suboptimal levels, awaiting natural selection to hitch subsequent fixed differences to achieve a better phenotypic optimum.

The study of tree sparrows entails one of the scenarios of how polygenic adaptation can drive rapid phenotypic changes during the early stage of highland adaptation. To further dissect the genetic constitution of adaptive response, we tested whether it is possible to discriminate between highland and lowland populations using top divergent markers (highly divergent SNPs with *F*_ST_ > 0.24) from 87 selected genes. Using phylogenetic reconstruction (i.e. the SNP phylogeny generated with SNAPP implemented in BEAST v.2.4.5) and PCA, we found that highly differentiated markers can indeed separate individuals from these two populations (e.g. using 956 highly divergent SNPs from the 87 PSGs as well as 158 highly divergent SNPs from 20 muscle related genes), Fig. 4c–d and Supplementary Fig. 10a–b). However, this separation was not observed in the SNP phylogenies generated from randomly picked SNP markers (Fig. 4e and Supplementary Fig. 10c). This suggests that even though the overall level of differentiation is not high, the top selective regions responsible for adaptive phenotypes can delineate highland from lowland individuals (PC1 = 37.4% of total variance and bootstrap score = 100%). If we assume a simple genetic trait whose phenotypic value is directly proportional to the number of selected alleles, we observed that individuals from the two populations also are rather disparate (*t* tests, *P* < 0.001, Fig. 4f and Supplementary Fig. 10d, Supplementary Table 27) when we counted the number of high elevation adaptive alleles across all the tree sparrows. Using this, we illustrate a possible scenario where multiple divergent loci can possibly drive the rapid evolution of phenotypes segregating strongly between highland and lowland sparrows. The polygenic nature might empower concerted changes across multiple loci leading to the large phenotypic differences even under the shallow genomic divergence [63–66].

## METHODS

### Sampling

All birds were caught with mist nets with permission from the Forestry Department of Qinghai and Hebei Province and in accordance with National Wildlife Conservation Law. Tissue collection procedures follow regulations of the animal experimental and medical ethics committee of the Institute of Zoology, Chinese Academy of Sciences. We sequenced, assembled and annotated the genome of a tree sparrow collected in Beijing. For comparative population genomics analyses, we re-sequenced the genomes of 23 individuals, of which eleven were collected from high elevation (3200–3213 m.a.s.l., Heimahe, Qinghai Province) and twelve from low elevation (60–100 m.a.s.l., Beijing and Hebei, Supplementary Table 14). We characterized cardiac muscle histology in an additional 15 individuals (seven highland and eight lowland individuals) and flight muscle histology in twelve individuals (six highland and six lowland individuals) (Supplementary Tables 28–29). Of the individuals sampled for muscle histology, six of these individuals were used in the cardiac muscle RNA-seq analysis (three from each group); eight were used in the flight muscle RNA-seq analysis (four from each group, Supplementary Tables 28–29). In addition, we collected ten lowland individuals for the hypoxia-exposure experiment (five for the experimental group and five for the control group). We used eight of these individuals for the cardiac and flight muscle histology (four from each group, supplementary Tables 28–29).

### Genome sequencing, assembly and annotation

#### Sample preparation and whole genome sequencing

For the tree sparrow for which the genome was sequenced (Fig. 1a), DNA was extracted from muscle tissue (frozen in liquid nitrogen) using the QiagenDNeasy Blood and Tissue Kit, following manufacturer protocol. Genomic DNA was sequenced using an Illumina HiSeq2000 platform. Libraries with different insert sizes were constructed at BGI-Shenzhen. To facilitate the assembly of the genome, we constructed different short-insert paired-end (170 bp, 500 bp and 800 bp) and mate-pair (2 Kb and 5 Kb) libraries. A total of 177 Gb of high-quality sequence data were obtained (Supplementary Table 1). After filtering out low quality and duplicated reads, 141.46 Gb data were used for *de novo* assembly of the tree sparrow. We assembled the genome using SOAPdenovo v2.04 (-K 31 and default parameter) [67] and SSPACE v.2.0 (-K 31, −x 0 -m 32 -o 20 -t 0 -k 5 -a 0.7 -n 15) [68]. The gene set was predicted by integrating *de novo* gene prediction, homology-based comparison and RNA-seq-based methods. Gene functions were annotated using Blastp based on their highest match to proteins in the SwissProt, TrEMBL databases (Uniprot release 2011–01), Gene Ontology [69] and KEGG database (Release 58) [70]. See supplementary methods for a detailed description of genome assembly and annotation (supplementary note 1).

### Population genomics

#### Sequencing strategy, quality checking and filtering

Genomic DNA from 11 highland individuals and 12 lowland individuals was extracted from muscle samples. All samples were sequenced on an Illumina HiSeq2500 platform at Science for Life Laboratory (National Genomics Infrastructure) in Sweden. Libraries of DNA (500 bp) were constructed according to the manufacturer’s introductions (Illumina). To avoid reads with artificial bias in the process of library construction and sequencing (i.e. low-quality reads, which mainly result from base-calling duplicates and adapter contamination), we carried out quality control and filtered out sequences according to the following criteria: (i) reads with ≥10% unidentified nucleotides (N); (ii) reads with >10 nt aligned to the adapter sequence, allowing ≤10% mismatches; (iii) reads with >50% bases having Phred quality <5; (iv) putative polymerase chain reaction (PCR) duplicates generated by PCR amplification in the library construction process (i.e. two paired-end reads were the same). A total of 483.75 Gb high-quality paired-end reads were retained for subsequent analyses (Supplementary Table 14).

#### Read mapping and SNP calling

After quality control, the paired-end reads were mapped to the tree sparrow genome using BWA v0.7.17 [71]. We initially performed single-nucleotide polymorphism (SNP) calling using two variant discover programs: GATK v3.7 [72] and Samtools v1.2 (http://www.htslib.org/). The overlapping SNPs called by both methods were used as the set of known SNPs for the first round of variant calling. Variant quality score recalibration, a postdiscovery error modeling algorithm implemented in GATK, was then used to further improve SNP calling. We filtered SNPs using VCF tools [73] and GATK using the following criteria: (i) minimum coverage = 138 (i.e. average of 6 reads per site per individual), (ii) root mean square (RMS) mapping quality ≥ 20; (iii) the distance of adjacent SNPs ≥ 5 bp; (iv) the distance to a gap ≥ 5 bp; (v) read quality value ≥ 30.

#### Population structure analysis

To estimate population structure, we removed all SNPs with a minor frequency (MAF) ≤ 0.1 and were not genotyped in >10% individuals. In order to avoid artifacts caused by tightly linked markers, we used PLINK v1.07 to calculate pairwise LD and removed one SNP from each pair of loci located within 20 kb of one another whose *r*^2^ is greater or equal to 0.5. We also removed SNPs deviating from Hardy-Weinberg equilibrium (*P* value ≤ 0.01). We used all of the remaining SNPs (6.47 M) to infer population structure for the tree sparrow. To estimate phylogenetic relationships, pairwise genetic distances were calculated among all individuals and a neighbor-joining (NJ) tree was generated using PHYLIP v3.695 (http://evolution.Genetics.Washington.edu/phylip.html). We performed a principal component analysis (PCA) using the smartpca program of EIGENSOFT [74]. Population genetic structure was inferred using FRAPPE v1.1 [75] and ADMIXTURE. We initially set predefined genetic clustering to K = 1–5 to explore potential population division between highland and lowland populations. Analyses were run with 10 000 maximum iterations.

#### Demographic history

We used FASTSIMCOAL v2.6 [38] to infer demographic history of the tree sparrows. We randomly picked 75 genomic regions from intergenic regions (0.5 Mb for each segment) and generated a two-dimensional–folded site frequency spectrum (SFS) using the doSaf function within ANGSD v0.917 [76]. We compared eight demographic models: the first two tested a single population hypothesis (M1 assumed a constant population size and M2 allowed population size to change over time), and the remaining six tested a colonization hypothesis. In the first three colonization models, we used the lowland population as a source population, assuming that all individuals of the highland population had migrated to the QTP at the same time, with unidirectional gene flow from lowland to highland (M3), unidirectional gene flow from highland to lowland (M4), and bi-directional gene flow between highland and lowland populations (M5, Supplementary Fig. S3). In the remaining three models, we examined similar demographic hypotheses while allowing for population size changes over time (M6–M8). All parameters were selected from a uniform distribution. For every demographic inference, we ran two separate analyses with each running seventy replicates. For each model with each replicate, we set 200 000 coalescent simulations with a minimum (−n) of 20 and maximum (–N) of 80 cycles in a conditional maximization algorithm. We specified a mutation rate of 3.3e^−9^ per site per generation following estimates for passerine birds [34]. We checked the consistency of likelihoods between the two analyses using *t* tests (Supplementary Table 30) and chose the run with the highest probability. We assessed the fitness of different models by comparing residuals between the observed SFS and expected SFS (Supplementary Fig. S11). We used the Akaike information criterion (AIC) to evaluate which model had the higher likelihood (Supplementary Table 15). For 95% confidence intervals (CIs), we simulated 100 replicates of the SFS from the *_maxL.par file (i.e. the parameter estimates that produced the maximum likelihood) for the best-fit run (minimized difference between maximum estimated likelihood and maximum observed likelihood) of the best-fit model. We performed the twenty replicate analyses described above for each of the 100 newly simulated SFS files. Finally, we calculated mean parameter estimates and 95% CIs from the 100 best-fit bootstrap replicates (i.e. using the best-fit run for each of the 100 simulated SFS files).

### Muscle histology

#### Flight muscle histology

Histological measurements were collected from highland tree sparrows (n = 6) and lowland tree sparrows (n = 6, Supplementary Table 28). Samples of the *pectoralis major*, the largest muscle in birds and the dominant muscle in the flight apparatus, were taken halfway along the length of the sternum, 3–5 mm lateral to the keel. Samples were coated in mounting medium then flash frozen in liquid nitrogen cooled isopentane. Muscle was sectioned (10 μm) along the muscle fiber length with a −20°C Cryostat (Microtome, Leica CM900, Germany). Three general types of fibers are recognized as components of avian skeletal musculature throughout the literatures, slow oxidative fiber, fast glycolytic fiber and fast oxidative fiber, the latter of which often comprises the entirety of the *pectoralis* muscle of small-bodied birds [43]. Muscle fiber types were identified by staining for succinate dehydrogenase activity (concentrations in mM: 0.6 nitroblue tetrazolium, 2.0 KH_2_PO_4_, 15.4 Na_2_HPO_4_, 16.7 sodium succinate) for 1 h at room temperature. In addition, to confirm that only fast oxidative fibers were present in the flight muscle of the tree sparrow, we also performed a myosin-ATPase staining in assay buffer (pre-incubated at pH 4.6, concentrations in mM: 100 sodium acetate, 10 EDTA, 200 tris, 18 CaCl_2_, 2.7 ATP, 1% CaCl_2_, 2% CoCl_2_, 2% ammonium sulphide). We used alkaline phosphatase activity to identify muscle capillaries by staining for 1 h at room temperature (assay buffer concentrations in mM: 1.0 nitroblue tetrazolium, 0.5 5-bromo-4-chloro-3-in-doxyl phosphate, 28 NaBO_2_, 7 MgSO_4_; pH 9.4). Images of microscope slides were taken using light microscopy and analyzed in Image J. Muscle fiber characteristics and capillaries were assessed following methods described in Scott *et al*. [30] and Scott and Johnston [77]. Stereological quantification methods were used to make unbiased measurements [78,79]. A sufficient number of images (>8) for each sample were analyzed to account for heterogeneity, which we determined by the number of images required to yield a stable mean value. Data are presented as means and standard errors. Sample and group means were compared using general linear model (LGM) in SPSS. Differences were taken as significant at *P* < 0.05.

#### Cardiac muscle histology

Fifteen birds were used for cardiac muscle histology (highland birds n = 7, lowland birds, n = 8, Supplementary Table 29). The left and right ventricles were dissected from the heart and subsequently fixed in 4% formaldehyde. Samples were sliced (5 μm) using Leica RM2235 paraffin slicer. Cardiac fibers were visualized by HE staining, and capillaries were stained with a mouse anti-CD34 antibody used at 1:100 (SGB-BIO, ZM-0046). Myocyte diameter of cardiac fibers and capillary density were measured from all images that were taken by a Leica DM750 light microscope. Data are presented as means and standard errors, and difference was determined with a significance level of *P* < 0.05. Myocyte diameters and capillary density were first compared between the left and right ventricles within highland and lowland tree sparrows using pairwise *t* tests. As we found no significant difference in either myocyte diameter or capillary density between ventricles (Supplementary Table 31), we pooled data from the left and right ventricles for subsequent comparisons.

### Acclimation experiment

Lowland tree sparrows were kept in an aviary for 31 days and were subsequently placed into individual cages. Ten individuals of similar weights (approximately 18 g) were selected and randomly divided into two groups of five individuals each. The control group was treated as before for 30 days, and the experimental group was exposed to normobaric hypoxia for 30 days using a hypoxic chamber with 14% of the oxygen content (simulating the oxygen concentration at 3200 m.a.s.l., 70% of ∼20%, latter of which is the content of oxygen content at sea level). All animal protocols were conducted in accordance with the Institutional Animal Care and Use Committee of Institute of Zoology. Control and experimental birds were sacrificed after 30 days and flight muscle and cardiac muscle were collected for the muscle histology. Muscle fibers and capillaries were stained, measured and compared using the same methods as described above.

### Identification of genomic regions under selection

To detect signatures of selection associated with colonization of the QTP in highland tree sparrows, we measured genome-wide variation (*F*_ST_ statistics and θπ values) between highland and lowland tree sparrows using 50 kb sliding windows using VCF tools. We generated 2000 (50 kb) simulated data sets under the inferred demographic history of two population and obtained null distributions of nucleotide polymorphism values using FASTSIMCOAL and ARLSUMSTAT. To obtain a site-based *F*_ST_ distribution, we generated 2000 gene genealogies with SNAPP implemented in BEAST v2.4.5 [80] and used GppFst [81] to produce a posterior predictive distribution of *F*_ST_ (for each gene genealogy we generated 410 *F*_ST_ values, roughly consistent with average SNP number in each 50Kb window). We subsequently calculated site-based *F*_ST_ of empirical data using Vcftools and picked the highly divergent SNPs (highly divergent [HD]-SNPs, using top 1% quantile of the simulated *F*_ST_ distribution, i.e. *F*_ST_ > 0.24). We identified outlier regions as the first 1000 windows containing the largest number of HD-SNPs (HD-SNPs ≥ 13, 5% outlier of the empirical distribution). We calculated nucleotide diversity (θπ, pairwise nucleotide variation as a measure of variability) in highland and lowland populations, respectively, and identified potentially selective regions as the regions showed decreasing level in the highland population (θπ_lowland/θπ_highland, 1.06, 5% quantile of simulated distribution). Considering linked selection driven by strong purifying selection, which could also lead a high relative genetic divergence (*F*_ST_), we used a combination of *F*_ST_ and shift in genetic diversity (the ratio of θπ values) between the highland and lowland populations to identify genomic regions that are under the selection. We hypothesized that positive selection will lead to a drop in genetic variability in the highland population comparing to the lowland population. Combining these two criteria, candidate regions often have higher genetic variability in the lowland population, but reduced variability in the highland population. Regions with strong background selection will often have reduced variability in both lowland and highland populations and will not pass our filtering criteria.

We also used Sweepfinder, a method based on SFS, to scan for selective sweeps, considering the spatial distribution of allele frequencies with composite likelihood ratio statistic [82]. We applied a range of thresholds based on the empirical distribution (top 1%, 2% and 5% of quantile) to detect regions with significant signatures of selection. We used KOBAS v3.0 to annotate and enrich these genomic regions with chicken genome as reference. We employed a binomial distribution correction for false discovery rate (FDR) to test if enriched gene functions were significant [83]. To test if genes related to muscle processes were overpresented in highland tree sparrows, we compared muscle related genes in the genome background as well as in the selected gene list with a χ^2^ test. In order to get an empirical estimate of the number of muscle related genes across the genome, we took all genes from muscle related GO terms (630 genes). Of the 87 candidate genes under the divergent selection, 20 were related to muscle processes (Supplementary Table 21), which were compared to the muscle related genes in the genome background (630 muscle related genes out of 16 925 genes).

### Transcriptome comparisons

To explore transcriptional changes with highland colonization, we performed RNA-seq analyses on the flight and cardiac muscles of the highland and lowland tree sparrows. Approximately 20ug RNA was extracted from six individuals for cardiac muscle and eight individuals for flight muscles (Supplementary Tables 25–26). Libraries were constructed according to the manufacturer’s protocol (Illumina). RNA sequencing was performed based on 150 bp paired-end reads using an Illumina HiSeq 4000 platform. After filtering low-quality, adapter-contaminated, and N-rich reads (>10%), the cleaned reads were mapped to our annotated genome of the tree sparrow and gene expression intensity was calculated using Kalliso in DESeq v1.22.1 [84]. Both DESeq and edgeR were carried out and a threshold value of fold change of two and a *P* value of 0.01 were used to detect DEGs between highland and lowland tree sparrows.

### Association tests of differentially transcribed genes with muscle phenotypes

To explore if the transcriptional changes of genes in the RhoA/ROCK signaling transduction are strongly correlated with muscle phenotypes, we correlated levels of gene expression of DEGs with muscle phenotypes in the highland and lowland individuals (eight individuals for flight muscle and six individuals for cardiac muscle). Muscle phenotypes are highly correlated and we used principal component analyses to summarize the patterns of trait changes (using the first eigenvectors from PCA that summarize 90% and 82% variation of three flight and two cardiac muscle phenotypes, respectively). We used each PC to calculate the loading score for each individual. Expression profiles (TPM, transcripts per million) of DEGs were log transformed. Principle component analysis loading scores for lowland and highland individuals were correlated with their TPM values of DEGs. We ranked the correlation coefficient and used a cutoff of *P* < 0.05 to select the genes with the strongest correlation with muscle phenotypes.

### PCA, phylogenetic trees and adaptive allele distribution of the selected genes

To test if highland tree sparrows can segregate from lowland tree sparrows in the putatively selected regions, we picked the highly divergent SNPs (*F*_ST_ > 0.24) from 87 selected genes (956 HD-SNPs) and 20 selected genes related to muscle functions (158 HD-SNPs). We randomly selected 956 and 158 SNPs from genome to generate phylogenies as comparisons (four NEX data sets can be found in Supplementary note 3). We run phylogenetic trees of these SNPs in the SNAPP with the default setting [85]. SNAPP was run for 110 000 generations with sampling every 1000 generations. The first 10 000 generations were removed as burn-in and maximum clade credibility trees were generated using TreeAnnotator within BEAST v2.4.5 [80]. We also performed principal component analyses of these SNPs in the smartpca program of EIGENSOFT.

We evaluated if the adaptive alleles of these SNPs distributed disparately between highland and lowland tree sparrows. The major alleles of the highland tree sparrows (AF > 0.5) were considered to be adaptive alleles of the high elevation (926 and 152 adaptive alleles in 87 candidate genes and 20 muscle genes, respectively). We counted the numbers of the adaptive alleles across all the highland and lowland tree sparrows and compared their distributions using *t* tests.

## Supplementary Material

nwz138_Supplemental_Files

## Data Availability

Sequencing data for the Eurasian Tree Sparrows have been deposited in Short Read Archive under project number PRJNA417520 and accession number SUB3203989. ***Conflict of interest statement**.* None declared.
