# Supplementary material for: Rapid phenotypic evolution with shallow genomic differentiation during early stages of high elevation adaptation in Eurasian Tree Sparrows
Source: Natl Sci Rev. 2019 Sep 12;7(1):113–27. doi: 10.1093/nsr/nwz138 (PMC8289047; doi:10.1093/nsr/nwz138)
Supplement: nwz138_Supplemental_Files [file nwz138_supplemental_files.zip › NSR_MS-2019-077.R1_tree_sparrow_Supplement.docx]

Supplementary materials

Rapid phenotypic evolution with shallow genomic differentiation during the early stage of high elevation adaptation in Eurasian Tree Sparrows

Yanhua Qu^1^*, Chunhai Chen^2^, Ying Xiong^1,3^, Huishang She^1,3^, Yong E. Zhang^1,3,4^, Yalin Cheng^1,3^, Shane DuBay^5,6^, Dongming Li^7^, Per G. P. Ericson^8^, Yan Hao^1,3^, Hongyuan Wang^9^, Hongfeng Zhao^9^, Gang Song^1^, Hailin Zhang^2^, Ting Yang^10^, Chi Zhang^2^, Liping Liang^2^, Tianyu Wu^2^, Jinyang Zhao^2^, Qiang Gao^2^, Weiwei Zhai^1,4,11^, Fumin Lei^1,3,4^*

1. Key Laboratory of Zoological Systematics and Evolution, Institute of Zoology, Chinese Academy of Sciences, Beijing, 100101, China
2. BGI Genomics, BGI-Shenzhen, Shenzhen 518084, China
3. University of Chinese Academy of Sciences, Beijing, 100049, China
4. Center for Excellence in Animal Evolution and Genetics, Chinese Academy of Sciences, Kunming, 650223, China
5. Committee on Evolutionary Biology, University of Chicago, Chicago, IL 60637, USA
6. Life Sciences Section, Integrative Research Center, Field Museum of Natural History, Chicago, IL 60605, USA
7. Key Laboratory of Animal Physiology, Biochemistry and Molecular Biology of Hebei Province, College of Life Sciences, Hebei Normal University, Shijiazhuang, 050024, China
8. Department of Bioinformatics and Genetics, Swedish Museum of Natural History, PO Box 50007, SE-104 05 Stockholm, Sweden
9. College of Life Sciences, Shaanxi Normal University, Xi'an, 710119, Shaanxi, China
10. China National GeneBank, BGI-Shenzhen, Shenzhen 518120, China
11. Human Genetics, Genome Institute of Singapore, A*STAR, Singapore, 138672, Singapore

*Corresponding authors: Y.Q. (email: [quyh@ioz.ac.cn](mailto:quyh@ioz.ac.cn)) or to F.L. (email: leifm@ioz.ac.cn)

1. Supplementary note 14
2. **Supplementary note 28**
3. **Supplementary note 311**
4. **Supplementary Figures**46
   1. Supplementary Fig.146
   2. Supplementary Fig.247
   3. Supplementary Fig.348
   4. Supplementary Fig.449
   5. Supplementary Fig.550
   6. Supplementary Fig.651
   7. Supplementary Fig.752
   8. Supplementary Fig.853
   9. Supplementary Fig.954
   10. Supplementary Fig.1055
   11. Supplementary Fig.1156
5. **Supplementary Tables 57**

4.1. Supplementary Table 157

4.2. Supplementary Table 258

4.3. Supplementary Table 359

4.4. Supplementary Table 460

4.5. Supplementary Table 561

4.6. Supplementary Table 662

4.7. Supplementary Table 763

4.8. Supplementary Table 864

4.9. Supplementary Table 965

4.10. Supplementary Table 1066

4.11. Supplementary Table 1167

4.12. Supplementary Table 1268

4.13. Supplementary Table 1369

4.14. Supplementary Table 1470

4.15. Supplementary Table 1571

4.16. Supplementary Table 1672

4.17. Supplementary Table 1773

4.18. Supplementary Table 1874

4.19. Supplementary Table 1975

4.20. Supplementary Table 2076

4.21. Supplementary Table 2177

4.22. Supplementary Table 2279

4.23. Supplementary Table 2380

4.24. Supplementary Table 2481

4.25. Supplementary Table 2582

4.26. Supplementary Table 2683

4.27. Supplementary Table 2784

4.28. Supplementary Table 2885

4.27. Supplementary Table 2986

4.28. Supplementary Table 3087

4.28. Supplementary Table 3188

Supplementary note 1. Genome assembly and annotation

*Genome sequencing and assembly*

DNA was extracted from muscle using the Qiagen DNeasy Blood and Tissue Kit following the protocol provided by the manufacturer. The genomic DNA was sequenced by Illumina HiSeq2000 sequencing platform. Libraries with different insert sizes were constructed at BGI-Shenzhen. To facilitate the assembly of the genome, we constructed different short-insert (170 bp, 500 bp and 800 bp) and mate-pair (2 Kb and 5 Kb) libraries (supplementary Table 1).

*Data processing*

A total raw data of 177 Gb were obtained (supplementary Table 3) and low quality and duplicated reads were filtered out according to following criteria:

1) Filtered the reads for which N constitutes more than 2% (for short-insert libraries) and 5% or 10% (for mate-pair libraries) of bases or poly (A) structure reads, respectively;

2) Filtered low quality reads: short-insert library reads that have more than 40% bases with quality scores ≤7 and large-insert library reads that have more than 30% bases with quality scores ≤7.

3) Filtered reads with adapter contamination: reads with more than 10 bp aligned to the adapter sequence (allowing less than or equal to 3 bp mismatch).

4) Filtered short insert-size reads in which read1 and read2 overlap > 10 bp, allowing 10% mismatch, where read1 and read2 are both ends of one paired end reads. If read1+read2+30 > insert size, the short insert-size reads were not filtered;

5) Filtered PCR duplicates: when read1 and read2 of two paired end reads were identical, these reads were considered as duplicates.

*Error correction*

For deep sequencing, the correct K-mers appear multiple times in the reads set, while random sequencing error-containing K-mers have low frequency. We used K-mer frequency information to correct the short-insert size (170 bp, 500 bp and 800 bp) libraries data. We chose K = 17 bp (because 417 = 16 Gb), which is larger than the genome size; thus, error-containing 17-mers were unlikely to exist in the genomes of the Eurasian Tree Sparrow. We built a hash table to store the frequency of all the 17-mers. Subsequently, for each read, we started from high-frequency regions and extended both sides to infer potential erroneous sites of low-frequency (<10) 17-mers. For each inferred erroneous site, we tested the impact of changing it to the other three allele types, and these changes were picked up as candidates if all 17-mers contained the allele that had a frequency >10. If we could not obtain any candidate that satisfied these criteria, we kept it; otherwise, the allele was revised to that with the highest 17-mer frequency. A dynamic programming algorithm was used to find the optimal solution with minimal changes. To increase speed, we used threaded parallelization to split read sets and handled them in parallel by sharing the same 17-mer hash table. After data filtering, we obtained 141.46 Gb for de novo assembly (supplementary Table 1).

*Estimate of the genome size with K-mer*

A K-mer refers to a sequence with K base pairs. After obtaining K-mers from the short-insert-size (<1Kb) reads with just one bp slide, frequency of each K-mer was calculated. The K-mer frequency fits Poisson distribution when a sufficient amount of data is present. The total genome size can be deduced from these data (Genome size = K-mer num / Peak_depth). We used 17-mer to estimate the genome size of the Eurasian Tree Sparrow, and the K-mer number was 45,334,919,774 and the Peak_depth was 38, so the genome size was estimated to be 1,193 Mb (supplementary Table 2).

*Genome assembly*

The genome was assembled using SOAPdenovo [1] via four steps: constructing contig, constructing scaffold, filling gap and constructing super-scaffold. Short insert libraries were split to construct de Bruijn graph, which was then simplified by removing tips, merging bubbles and solving repeats to get contigs. All clean reads were mapped onto the contigs to construct scaffolds. The clean data from short-insert size libraries were used to fill gaps. Finally, all clean reads were mapped onto the scaffolds to construct super-scaffolds by SSPACE [2]. Assembly quality and completeness were assessed by checking BUSCO analysis [3] and ESTs evidence. The final contig and scaffold sizes, N50 and N90, were calculated based on the minimum length of sequence. The sequencing coverage, depth, GC content distribution and heterozygosity rate of the assembled genome sequences were evaluated by mapping all sequencing reads of the short-insert-sized libraries back to the scaffolds using BWA [4].

Genome annotation

*Repeat annotation*

Tandem repeats were searched across the genomes using Tandem Repeats Finder software (version 4.04, http://tandem.bu.edu/trf/trf.html). Transposable elements (TE) were identified in the genome by a combination of homology-based and de novo approaches. For homology prediction, RepeatProteinMask and RepeatMasker (version 3.3.0, http://www.repeatmasker.org/) were used with default parameters against Repbase (release 16.03, http://www.girinst.org/repbase/). *De novo* prediction was performed as follows: the ab initio prediction program RepeatModeler and LTR-FINDER (version 1.0.5, <http://tlife.fudan.edu.cn/ltr_finder/>) were employed to build the *de novo* repeat library, then the contamination and multi-copy genes in the library were removed. Using this library as a database, RepeatMasker was run to find and classify the TE in the genome.

*Gene annotation*

A combination of *De novo* gene prediction, homology-based comparison and RNA-seq data was applied to annotate the genome of the Eurasian Tree Sparrow. In order to obtain a consensus gene set, all the predicted genes were integrated by GLEAN software (http://sourceforge.net/projects/glean-gene/). For the homology-based comparison, all available protein sequences of the Medium Ground Finch (*Geospiza fortis*), the Zebra Finch (*Taeniopygia guttata*), the Collared Flycatcher (*Ficedula albicollis*), the Ground Tit (*Parus humilis*), the American Crow (*Corvus brachyrhynchos*) and the Golden-collared Manakin (*Manacus vitellinus*) were downloaded from Ensembl and aligned to genome of the Eurasian Tree Sparrow using TblastN. Homologous genome sequences were aligned against matched proteins using Genewise [5] to define gene models. Augustus was used to predict coding genes. For RNA-seq based prediction, we mapped transcriptomic reads to the assembled genome using HISAT [6]. We combined HISAT mapping results together and applied StringTie [7] to predict transcript structures. Using Glean [8] and homolog approach to integrate the all predicted gene structures. We obtained final gene sets, of which 70% and 87% were annotated by Gene Ontology [9] and KEGG [10], respectively (supplementary Table 10). The tRNA genes were *de novo* predicted by tRNAscan-SE software, with eukaryote parameters on the repeat pre-masked genome. The rRNA fragments were identified by aligning the rRNA sequences using BlastN at E-value 1e-5. The snRNA and miRNA were searched by aligning and searching INFERNAL (version 0.81) against Rfam database (release 9.1).

*Non-coding RNA (ncRNA) annotation*

The tRNA genes were *de novo* predicted by tRNAscan-SE software, with eukaryote parameters on the repeat pre-masked genome. The rRNA fragments were identified by aligning the rRNA sequences using BlastN at E-value 1^e-5^. The snRNA and miRNA were searched by aligning and searching INFERNAL (version 0.81) against Rfam database (release 9.1). Finally, 1009 non-coding RNAs were identified in the Eurasian Tree Sparrow genome (supplementary Table 8).

*Gene functional annotation*

Gene functions were annotated using Blastp, based on the highest match to proteins in the SwissProt and TrEMBL databases (Uniprot release 2011-01). The motifs and domains in the protein-coding genes were determined by InterProScan (version 4.7) searches against six different protein databases: ProDom, PRINTS, Pfam, SMART, PANTHER and PROSITE. GO ID for each gene was obtained from the corresponding InterPro entry. All genes were aligned against KEGG database, and the pathways in which the genes are involved were derived from the matched genes in KEGG. Based on homology searches, approximately 82.35% of the protein-coding genes in the Eurasian Tree Sparrow were found to have homologues in the protein databases including Swissprot and TrEMBL or were classified into functional categories according to InterProScan, KEGG and GO.

References:

1. Li, H. et al. TreeFam: a curated database of phylogenetic trees of animal gene families. *Nucleic. Acids Res.* 34, D572–D580 (2006).
2. Boetzer, M., Henkel, C.V., Jansen, H.J., Butler, D. & Pirovano, W. Scaffolding preassembled contigs using SSPACE. *Bioinformatics* 27, 578–579 (2011).
3. Simao, F.A., Waterhouse, R.M., Ioannidis, P., Kriventseva, E.V. & Zdobnov, E. M. BUSCO: assessing genome assembly and annotation completeness with single-copy orthologs. *Bioinformatics* 31, 3210–3212 (2015).
4. Li, H. & Richard D. 2009. Fast and accurate short read alignment with Burrows–Wheeler transform. *Bioinformatics* 25, 141754–141760 (2009).
5. Birney, E., Clamp, M. & Durbin, R. Genewise and genomewise. *Genome Res.* 14, 988–995 (2004).
6. Kim, D., Langmead, B. & Salzberg S. L. HISAT: a fast spliced aligner with low memory requirements. *Nat. Methods* 12: 357–360 (2015).
7. Pertea, M. et al. StringTie enables improved reconstruction of a transcriptome from RNA-seq reads. *Nat. Biotechnol.* 33, 290–295 (2015).
8. Elsik, C. G., Mackey, A. J., Reese, J. T., Milshina, N. V., Roos, D.S. & Weinstock, G. M. Creating a honey bee consensus gene set. *Genome Biol.* 8, R13 (2007).
9. Ashburner, M. et al. Gene ontology: tool for the unification of biology. The Gene Ontology Consortium. *Nat*. *Genet.* 25, 25–29 (2000).
10. Kanehisa, M. & Goto, S. KEGG: kyoto encyclopedia of genes and genomes. *Nucleic. Acids Res.* 28, 27–30 (2000).

Supplementary note 2. Limitations of the acclimation experiment and the phenotype plasticity in other traits.

*Background*

To explore if the changes in muscle phenotype in the highland tree sparrows are a temporary plastic response to the high elevations, we experimentally exposed the lowland tree sparrows to an oxygen level corresponding to that of 3,200 m.a.s.l (Fig. 1). We focused on the oxygen level at high elevation because muscle phenotype changes have previously been observed in tree sparrows at high elevations [1], but not in the tree sparrows at high latitudes (i.e., a proxy for cold temperature). Thus, we considered that the low oxygen level is considerably more important selective pressure at high elevations than is low temperature.

*Results and limitations*

After experimentally having exposed lowland tree sparrows to hypoxic condition for 30 days, we could not observe muscle phenotype change similar to those observed in the highland sparrows (Fig. 1). While this result suggests that short-term plastic response does not contribute strongly to the observed muscle phenotypes in the highland population, there are some other factors not considered in the experiment that theoretically could also influence the muscle phenotypes.

Fig. 1. (a) Acclimation experiment protocol for the hypoxia-exposed lowland tree sparrows and control lowland tree sparrows. (b) The fiber areas of the flight muscle (left), the number of capillaries per flight muscle (middle) and the capillary density of the cardiac muscle (right) in the experimental birds (grey boxes) were similar to those of the control birds (white boxes) and the wild lowland birds (blue boxes), but reduced compared to those of the highland tree sparrows (orange boxes).

*Limitations of acclimation experiment*

1. The duration of the experiment period. Our experiment had to be limited to one month due to problem with keeping tree sparrows captive longer. This time may not have been sufficient to induce the same phenotype changes in lowland birds as those observed in birds living on the Qinghai-Tibet Plateau.
2. The acclimation conditions. Hypoxia alone may not have been a strong enough factor to induce the phenotype changes in these birds. It is possible that the combination of hypoxia and cold temperature constitutes a strong factor.
3. Muscle development. The study birds were all adult individuals. There is a possibility that muscles are more plastic in the early development stages in three sparrows.
4. Other factors, e.g., epigenetic effects, region-specific niche, and the food resource available, may potentially contribute to muscle phenotype changes.

Considering the uncertainties, we regard our conclusion that that muscle phenotypes are stable and do not respond the hypoxia acclimation as preliminary and conditioned given the experimental design.

*Phenotype plasticity in the hemoglobin level of the hypoxia-exposed lowland tree sparrows*

In contrast to no changes in the muscle phenotypes, we observed that other phenotypic trait, e.g. hematocrit level, increased significantly in the hypoxia-exposed lowland tree sparrows, suggesting that this physiological phenotype is susceptible to a plastic response (Fig. 2). When birds, mammals and humans of lowland origin ascend to high altitude, or are exposed to hypoxia acclimation experiments, they often respond to hypoxia by increasing hemoglobin and hematocrit concentration [2-7]. However, the elevated hematocrit and hemoglobin concentrations also increase blood viscosity, producing a higher peripheral vascular resistance that can compromise cardiac output [2-7]. Thus, instead, many species native to high elevations evolve genetic changes in hemoglobin structure that increases blood-oxygen affinity to avoid harmful increases in red blood cell concentration [8].

Fig. 2. Lowland tree sparrows increased hematocrit concentration after exposed to hypoxia condition, compared to control birds.

In the case of the highland tree sparrows, we observed hematocrit changes that are similar to the acclimatization response to hypoxia in lowland tree sparrows, suggesting that certain phenotype, like hematocrit level, is still under the plastic reaction. Thus, we may expect that the Eurasian Tree Sparrow in the early stages of highland adaptation were in an intermediate stage between plastic reactions and adaptive evolution, with certain traits showing plastic reaction while others were under adaptive evolution.

References:

1. Sun YF, Ren ZP, Wu YF, Lei FM, Dudley R, Li DM. Flying high: limits to flight performance by sparrows on the Qinghai-Tibet Plateau. Journal of Experimental Biology 219, 3642–3648 (2016).
2. Storz, J. F., Scott, G. R. & Cheviron, A. Z. Phenotypic plasticity and genetic adaptation to high-altitude hypoxia in vertebrates. *J. Exp. Biol.* 213, 4125–4136 (2010).
3. McGrath, RL & Weil, J. V. Adverse effects of normovolemic polycythemia and hypoxia on hemodynamics in the dog. Circ. Res. 43, 793–798 (1978).
4. Winslow, R. & Monge, C. Hypoxia, Polycythemia, and Chronic Mountain Sickness. Baltimore, MD: Johns Hopkins University Press (1987).
5. Monge, C. & León-Velarde, F. Physiological adaptation to high altitude: oxygen transport in mammals and birds. Physiol. Rev. 71, 1135-1172 (1991).
6. Connes, P., Yalcin, O., Baskert, O., Brun, J. F. & Hardeman, M. In heath and in a normoxic environment, VO2 max is/is not limited primarily by cardiac output and locomotor muscle blood flow. J. Appl. Physiol. 100, 2099 (2006).
7. Schuler, B., Arras, M., Keller, S., Rettich, A., Lundby, C., Vogel, J. & Gassmann, M. Optimal hematocrit for maximal excercise performance in acute and chronic erythropoietin-treated mice. Proc. Natl. Acad. Sci. USA 107, 419–423 (2010).
8. Zhu, X. et al. Divergent and parallel routes of biochemical adaptation in high-altitude passerine birds from the Qinghai-Tibet Plateau. Proc. Natl. Acad. Sci. USA 115, 1865–1870 (2018).

Supplementary note 3. Dataset used in the SNAPP phylogeny.

| **NEX file of 956 SNPs from 87 candidate genes** |
| --- |
| #NEXUS  Begin data;  Dimensions ntax=46 nchar=956;  Format datatype=binary symbols="01" gap=-;  Matrix  H-101_1 11111111111111111111001010000000110000000000000000000000000010111011000100100000000000000000011000100000000000000010100000000000000000000000000000100000000000001110000000111000000000000000000000000000000010010000000000000000000010000000000001010100001001000000000000000000000010000001100000000000000001100000000000010000010010000010000000000000000010000000000000000000110000100001000100000010000000000001000000011000010000001001110000000000000000000000100000010000010000000000000000001100000000000011000100000000001000000000001000000000000010000000000000000101000000000000000000000000001000000000000000100011011000000000000000000000000000000000000000000000000000000000000000010000000000000000000110000100000110000000000000000000010100110000000000000000001100000000000000101110000000010000000011010111111111000000000000001000000000000000000000001000000000000000001000010100000000000000110000000000100011000000000000000100000101100000000000001010100101000000  H-101_2 11111111111111111111001010000000110000000000000000001000011111111011000110100000000000000000011011111011001111100111100111111110000000000001010111100000000000001110000000111111101111010000011000000011101110011000000000010000001011000100111101010110001101011000111001011111010011100011100000000010010011111011111000010000010011110010110000000000000010000000000000010100110000101111000100000010010000000011000000011000010000001001110000011010010000000011111101010000010000000001000101111111111110111111100101000011101000010001001010101010001110001101100000010111111101111000000001111011101111011110011111100011011000000000000000000000000000011111111111110000011111111110100111011110101100001100100110000110001111000011100111110111010100111111111111111111001111000000000000101110000001111111111011110111111111111000111001001110000000000000000000001000000100000011001110011101100000000100111000100010101011000000000000000100000101110000000001101010111111000100  H-103_1 00001010001000000000000010000000010000000000000000000000000010111011000101011111111111111101101010000000001110000110111000000000000000000000000000100000000000001110000000111100000000000000000000000000000000000000000000000000000010000011110001000110000000011000000000011000000000000000000000000000000000000000000000010000000000000000100000000000000010000101111111010011001111010010000000100010010000000001000000000000000000001001110000000000000000000000000001010000000000000000000101001110000010001110000001000000001000000000000110001000000000000000000000000000100001000110000000000000001001010110000001011100111000000000000000000000000000000000000000011000000000000000100000010000100000000000000110000100000000000011100100000000001011000001001100010000001111000000000000000001111110000000000001010000000001000000000000000010000001000000000000000000000000010000101000000100000000000000010000000000001000000000000000000100000101100000000001101000110010000000  H-103_2 11111111111100001000000010000000110111111111111111101110000010111011000101011111111111111101111011000100001110000111111101110110000000000000001000100000000000001110000000111111011101011111100000100111101110010000000010010000001010000111111101011110000001011100000000011111110111111111100000000000000100111000000000010000010011110010101100000000000010000111111111010011001111011011001100100010010000001001000100111100110100001001110000000000000000000010000111110111111111111110111111111111111111011111100101110011111001111110000110001000111111010111101010111111100001010110000000001000011111110110010001111100111011100000000000000000000000011111111111111000000000000000101111011111101000000100111110001110001101001011111111000100011011000001001100010000111111000000000000100001111110000000011011110111100111111111001001011110000001011111111111111011000000011000111111010101110000001000011010100111111000000000000000000100000101110000000001101010110011100101  H-104_1 00000010010000001000010110000000110000000000000000000000000010111011000110100000000000000010011000000000000000000001000100000100000000000000000001000000000000001110000000000010010101001111100000000000100010010000000000000000000010000000000001010110010001000000111011001000000010100001100000000000000000000000000000010000000000000000000010111111111101101000000000000100110000101100000000001010010000001000010000011000010000001001110000000001000000000010000101110111111111111110111111111111101010011110000001000011001001101110000110001000111101010001100010001111100001010110000000000000001011010110010001100011011000000000000000000000000000000000000000000000000000000000000111010000101000000000100100010110011100011000000110011111001011000001000000010000111110000000000000100100000100000001011011110111101111110000000000000100000000000000000000000000000000000000001000010100000000000000010000000000000000000000000000000100000101100000000001101000110010000001  H-104_2 11000010011100001000010110000000110000111111111111000000000010111011000110100000000000000010011011100000000011111011101100110100000000000000010001100000000000001110000001111110010101111111100100000000100010010000000000000000000011001010100001011110010001101100111111111111100010100001100000000000000000000000000000010000000010000010100111111111111101101000011101000100110000101100000000011110011000001001110000011000010100001001110000011111101100000010110111110111111111111110111111111111101010011110000001000011001001101110000110001000111111010001100010001111100001010110000000000000001111010110010001100011011000000000000000000000000000011000000000001000011111110000000111011000101001000001111100010110011100011000000110011111001011000001000000010000111110000000000000111110000100010001011011110111111111110011010000000100000000000000100000001011000100000011001001010101000000000100110000100100111000000000000000010100000101100000000001101010111010000101  H-105_1 11111111111111101111000010000000110000000000000000000000100010111011000100000000000000000000011001000000000000000011000000000000000000000000000111000000000000001110000000000000101010001111100000000000000010010000000100000000000011100000000000000000011001000000000000000001100101000000000000000000000000000000000000010000000000000000100000000000000000000000000000000100110000100101000100100010000000000000010000011000010000001001110000000000000000000001100100000000000000000000000000000000000000000000000000100000100000010000000000000001000000000000000000000100100001010000000000000100001111110111110010100000000000000000000000000000000000000000000000000000000000000000000001011000000000000000000100000000000100011000010000001011010000000001001100010000000010000000000000100000010100000000011011110111100011010000001000000100000000000000000000001000000100000011001000010000000000000000000000000000001000000000000000000100000101100000000001101000110010000001  H-105_2 11111111111111111111011110000000111111111111111111001000100010111011000111111111111111111110011011111111111100000011101111111110000000000001000111100000000000001110000000100000101010011111100001100011101110010000000110000000000011110111110011101100011001111000000000011111110101011110000000000000000000000000000000010000000010000011100111111111111111101000000000000100110000100101001100100010000000000001110000011000010000001001110010011000000000111101100110011000000000000000000101011001111111111111100100110010111000110000001000000001000000101110000000000100100001010110011111111100001111110111111110111111111110000000000000000000000000011111111111111000010000000100000001011000001000000101011101011111111110011011111111111111011111110001001100010001111110100000000000101111111110010000011011110111100111111010011001001100000000000000000000001011000100000011001001011101111000000000111000000000101011000000000000000100000101111000000001101010111010110101  H-118_1 01000000000000000000001000000000110000000000000000001000000010111011000101011111111111111100011000011011111110000111000100000000000000000000010000000000000000001110000000100000101010000000000000000000100000010000000000000000000010000011110001001000001100010000000000000000000101000011100000000000000010000000000000010000000001110010000000000000000010000000000000010011001111010001000000001100100000000001000000011000010000001001110000000000000000000000100010010000010000000000111111111111101010011110000001000011001001101110000110001000111111010001100000000100000001010110000000000100001111110111111101111100111000000000000000000000000000000000000000001000000000000000000000011000001000000000000110000100001100011001100100000000001011100001000000010000000000000000000000100001111110000000011011010111111111110000000000000110000000000000000000001000000000000000001000010001000000000000000000000000000000000000000000000100000101100000000001101010110101100100  H-118_2 01110110011100001000001011000000110000100000110000001000100010111011000101011111111111111100011011111011111110000111101100000000000000000000010000000000000000001110000000111101101010011111100000000000100010010000000110010000001011011111111101011000001101010111000000011111100101000011100000000000000010000000000000010000010011110010110010011001100010000111111111010011001111011001000000101101100111111111000000011000010000001001110000000000010000000011101011010000010000000000111111111111101010011110000001000011001001101110000110001000111111010001100000000100100001010110000000000100001111110111111101111100111000000000000000000000000000000000000000011000000000000000000001011000101100000110111110000100001100011001100100000000001011100001000000010001000001000000000000110001111110010000011011010111111111110001011000011111111111100000000000001000000100000000001000010101000000000000111000100000111000000000000000000100000101101000000001101110111101110101  H-119_1 10000010001000000000010110000000000000000000000000001000011111111011000100100000000000000001111001000000000010000010000000000000000000000000000000000000000000000000000000011100101000010000000000000000100000010000000000000000000010011111111101010110011101001000000000001010000010100001100000000000000010000011100000010000000001000000000000000000000000000000000000000000000000001011000100000010000000000001000000011000010000001001110000011000000000000000000000110111111111111110000001000000010100100011111110000011001000000000000111111010011110000101100010001111100001000000000011111100011111110111111101110000100101100000000000000000000000000000000000000000000000000000000111010000000000000000100110000110000100000011100110011111010100011110110011101111001110000000000000101100111110000000011001110111111111101000000000000000000000000000000000000000000000000000000000000001000000000000000000000000000000000000000000000100000101110000000001101000110010000100  H-119_2 11111110111111111111110111111111110000000000000000001000011111111011000100100000000000000001111001100100010010000111101111111110000000000000001000111000000000001111111111111111101010011111101010000000100010010000000100010000001011011111111101010110011101111100100000011110010010100001100000000000000010000011100000010000000011110011110011111111111111101111111111010111111111111011000100000010010000000001010100011000010100001001110000011001010000000011010011110111111111111110000001000000010100100011111110000011001000000000000111111010011110000101100010001111100001000000000011111100011111110111111101111100100101100000000000000000000000000000000000001000011111111000110111010110101100000001110110000110001100000011100110011111010100111110110011101111001110000000000000111110111110000000011001110111111111101001011000010111111111111111111111111010000011011011111111010101000000000000011100000001110011000000000000000100000101110000000001101100110010000100  H-120_1 00000000010100000000000010000000010000000000000000001000000010111011000101011111111111111101101011100000000010000010000000000000000000000000000001011000000000001110000000111100101010000000000000000000100010010000000010000000000010011011111001011110000001000000000000000010000010100001100000000000000011100010000000010000000001000010100000000000000010000000000000000000110000100011000000000000000000000001000000011000010000001001110000000000000000000000000000110111111111111110000101011100010110001111100101000011001001001110000011110001000000000100011000110000100001000000000000000100011110010110000010010100111000000000000000000000000000000000000000011000000000000000000001010000100000000110001110000110000011011011100101000011010100111110110011101111011010000000000000100001111110000000011011110111100001110000001000000000000000000000000000000000000000000000000000000000000000000000011000000000000000000000000000000100000000010000000001001010110001000000  H-120_2 00000010010100001000000010000000110000000000000000001000100010111011000101011111111111111101101011111011111110000111000101110110000000000001010111111000000000001110000000111110101010011110110000000000100010010000000010010000000011011111111101011110000001110100100100001011110010100001100000000000000011100010000000010000010111000010100000000000000010000111111111010100110000100011000000111111101000000001010100011000010000001001110000011000000000000011011111110111111111111110000101011100010111001111100101000011001001101110000011110001000000000110011000110000100001000000000000000100011110010110000010011100111000000000000000000000000000011111111111111000000000000000010111010110101100000110101110000110000011011011100101000111010100111110110011101111011010000000000000110011111110000000011011110111100001110000001000011111000001000000000000001011000100000011101111010100110000000000011000000000111000000000000000000100000101111000000001101010110111110101  H-121_1 00000011010100000000000010000000111000000000000000001000000010111011000100100000000000000010011001100000000000000000100100000000000000000000000000100000000000001110000000100000000000010100000000000000100010010000000000000000000011000111110001000110001101000000000000010000000011100001100000000000000010000000001000010000000000000010100000011000000010000111111111000000000000000101000100000000010000000000000000011000010000001001110000000000000000000001100000010000000000000000011101000000010110001111100001000011000000110000000010000000000110000100000011000000011100100000001011111000001111110111010010100011011000000000000000000000000000000000000000011000000000000000000001010000000000000000000110010110010010011011000110011111011010000001001100010000011110000000000000101100000110000000011001110111111111111011001000000000000000000000000000000000000100000011001000001000000000000000010000000000100000000000000000000000000101100000000000001000000000000100  H-121_2 11111111111100001000000010000000111000000000000000001000000010111011000100100000000000000010011011100000101110000101100100101000000000000001011110111000000000001110000000111111111111011110100000000000100010010000000000000000000011100111111101010110001101110100000000011000010011100001100000000000000010000000001000010000000011000010100001111111100011010111111111010111111111111101000100011111110000001010000000011000010100001001110000000001010000000001110111010000000000000000011101000000010111001111100101000011000000110000000010001000000110000100000011000000011100100000001011111000001111110111110010100011011000000000000000000000000000000000000000011000001111110000100001011110000001001110000110010110011010011011000110011111011011000001001100010000011111000000000000111110000110000000011001110111111111111011001001000100000000000000000000001000000100000011001000011101110000000000111010010110100011000001110111111111100101110000000001101111110111100101  H-122_1 00100000000000111111010110000000110000000000000000000000011111111011000110100000000000000001101010111011110000000101000001110110000000000000000000111000000000000000000000100000101010000000000000000000100000010000000000000000000010000000000001111100011100000000000000001010010100001110000000000000000010100000000000010000000010000000000000000000011101101000000000010000110000100011000100000010000000000001000000010000010000001001110000011000000000000000010000010000010000000000000001000000010100100001111110111100111000000000000111111010011110000101100010001111100001000000000011110100011111110111111110100011011000000000000000000000000000000000000000011000000000000000100111010000000000000000000100010110011010011011100110011111000101000001001100010000001111100000000000101110111110000000000001010111111111011000000000010100000000000000000000001000000000000000001000010000000000000000011000000000010000000000000000000100000101100000000001101110110010000000  H-122_2 11101111111111111111010110000000110000000000000000000000011111111011000110100000000000000001101011111011111110000111100001110110000000000001010111111000000000001111111110111101101010010010000000000000100010010000000000010000001011000000000001111100011101011000100000011110010101011110000000000000000010100000000000010000000011000010000011111111111101101111111111010000110000100011000100100010010000001011000000011000010000001001110000011000010000000011110111010000010000000000000001000000010101100001111110111100111000000000000111111010011110000101100010001111100001000000000011111100011111110111111110100011011000000000000000000000000000011111111111111011100000000000100111011110000100000000111100010110011010011011100110011111000101000001001100010000001111100000000000111110111110010000000001010111111111011010011000010110000000000000000000001011000100000011001001011001000010000000111000110000010111000000000000000100000101111000000001101110111111100100  H-123_1 00000000000000000000011010000000010000000000000000000010011111111011000100100000000000000000001000000100000010000000100100000000000000000000010000000000000000000000000000000000100010010000000000000000000000010000000000000000000010000000000001010000000001001000000000011111000010100001100000000000000010000000000000000000000000000010000000000000000000000000000000000000000000000010000100000010000000000001000000011000010000001001110000000000000000000000000000010000000000011100000101000000011100100011100100010001001000000000001001110010010111010000000000110101100000010000011101110000001111110111111101100011111000000000000000000000000000000000000000001000000000000000000000010000001000000000000110000110000010011000100010000000000100111110110011101110111110000000000000100110111110010001010001010111101101111010000000000000000000000000000000001000000100000011001000010000110000000000000000000000000000000000000000000100000101110000000001101000110000000000  H-123_2 00000010010100001000011010000000110000000000000000000010011111111011000100100000000000000000001011000100001110000110111100001100000000000001010001000000000000001111111111111101101010011111100010011111101110010000000000000000000011011111111101010000000001111000000000011111100010100001100000000000000010000000000000111000000110000010110111111111111111111000000000010111111111110010000100000010010000001011110000011000010000001001110000000000010000000011101110010000000000011100000101000000011100100011100100111001001000010000001011110010011111010000000000110101100001010000011111111000001111110111111101100011111000000000000000000000000000000000000000011000011111110000000001011000101000000111100110000110000010011000100011000000000100111110110011101110111110000000000000111110111110010001010001010111101101111011001001001100000000000000000000001011000100000011001001111001110010001100010001010111110011000000000000000100000101110000000001101110110110110101  H-201_1 00000010011000010000000010000000001000000000000000001000000010111011000100100000000000000000011001100100000000000000000100000000000000000000010000000000000000001110000000111100000000000000000000000000100010010000000000000000000010000000000000010100011000000000000000000000000010100001100000000000000010000011100000010000000000000010100000000000000010000000000000000100110000100011000100000010000000000011000000011000010000001001110000000000000000000001100010010000010000000000111111100000010100100010100000000011001000010000000000000010100000010000000000110101011100100000001101111100001110000001110001100011011000000000000000000000000000000000000000001000000000000000000000011000100000000000010100001001100110000000000110010111000101000001001100010000011110000000000000100001111110010000011011010111111111110010000000000100000000000000000000001000000100000011001001000101000000000000010000000000001000000000000000000000000101100000000000001010111000000100  H-201_2 11110111111111111111000010000000001000000000000000001000000010111011000100100000000000000000011011100100000000000011011110000000000000000000010111111000000000001110000000111111111111001110100000000000100010010000000000010000001011000100011111110100011001001000111111011110000010100001100000000000000010000011100000010000000000000010100100000010000010000000100000101100110000100011000100010010010000001011010000011000010100001001110000011001000000000001100011010000010000000000111111100000010100100011100100000011001000010000000000000010100000010000000000110101011100100000111111111100001110001001110001100011011000000000000000000000000000001111111111101000011111111000100111011000111100000101111100001001100110000000000110011111010111000001001100010000111110000000000000100001111110010000011011010111111111110011000001011100000000000000000000001011000100000011001001011101011000000000011001101010101000000000000000000100000101111000000001101110111111110101  L-108_1 00000000000000000000001010000000011000000000000000010000000010111011000110100000000000000010011001000100000001111001101100001000000000000000100000000000000000000000111111000000000000001111100000000000000000000000000000000000000001000000000000100001100000000000000111000001100101011110010000000011101101111111100000000000000011000010100000000000000010010000000000000100110000100100111100010000000000001000010000010000000000001001110011011001000000000001100010000000000000000000000101000000010100100011100100000001000000010000001010000101000000000100000100110000011110101000111111111011100000010110000010011100111011111111111111011111111111100000000000010000000000000000000000100000101000000111000101001001101101011000011001100000001011000001001100010000001011000000000000001100111110010001010001010111101101110000001000010111111111111111111111110000000000000000000000000000100000000000011011011110000011000000010010110001100001100000000000001000110010110000  L-108_2 00000010001100000000101010000000111000000000100000010011100010111011000110100000000000000010011001000100000001111011111110001001111111111110101000000000000000000001111111000010111101111111100000011111111111111111111111101111110111000000010011110001100000000011111111101001100101011110011100000011101101111111100011111100010111110011111100000000000010010000000000000100110000100100111100011111101111111101110000011111111100001001110011011001000000000001101010000000000000000000000101000000010100100011100100000011000010010001001010000101000000000100000100110000011110101000111111111011100000010110000010011100111011111111111111111111111111100000000000010011100000001111011000100001111000001111000101001001101101011000011001100000001011000001001100010000011011000100110000001110111110010001010001010111101101111000001110110111111111111111111111110100111010011011111110100010110000001100011111011110110111111111111111111011111101111000000001111000110010110000  L-110_1 00000000000000000000001001110111000111111111111111000100000000000000000000100000000000000010010001000100000000000001101110001000000000000000000000000000000000000001111111000010010101011111100000100111001100000000000110000000000001000000000010100000000000000000000000000000000101010010000010001011101001110000000000010000000011000000100000000000000010000000000000000100110000100000000000000000000000001000000000000000000000000000000000011000000110111000100000010000000000000001000000000000010100100011100100100000000000000000000010000001000000000100000000110000000000001000000000000000000000000000000010011100100000000000000000000000000000000000000000010000000000001000000000100000101000000101000100000110001100000000000101100000001011000001001100010000000001000000000000000010000000010001000001010111100001001100101000010100000000000000000000000000000000011000000110000000110000000000011001010000000011111111110111111010000000000111111100001000000010000000  L-110_2 01000010000000000000111001111111000111111111111111000110100010111111111110100000000000000010011111000100000001010011101110001001111111111110110000100000000000000001111111000010010101111111111111111111011101111111111111101111110101100000000010100000000011000011111111101001100101010010000010001011101011111111100000010000010111111010100100000000000010000000000000000100110000100111111011011111101000001001111111111111111111111111111100111111101111111111100111010000000000000001000000000000010101100011111110111101111010010001100010000001001110001110011101110001111101101000011111111111101111011110010010011100111101111111111111111111111111100000000000011111100000001111011001100001101101001111000110001110001101000100001101100000001011000001001100010000001111100000000000101110000101111111111011110111111111111110101000110111111111111111111111111000000011011000111110010001111010001100011111011110100011111111111111111111111111111111111111111001110010110100  L-111_1 00000000000000000000000010000000111111100000000000111000000000000100111010100000000000000000010101000100000000000000100110000000000000000000001000000000000000001110000000000010010101011110100000000111001100000000000110000000000000000000000001000001101000000011111111100001101101011110011111110110011011101011111000000000010011110000100000000000000010010000000000000100110000100100111010011000001111111100010000001000010011111001110010000000000000000001101010010000000000000001000000000000010100100011111110111100100100010000011000000001000000101110011101010000100001001000101111111011101000001000000001011100100101100000000000110000000000000000000000011000000000000000000000000000101101001100000100100100001101100100001001100000001011000001001100010000000001111111111111001110000001111111100100001000000000001100001000010111111111111111111101110100000010000000110000010000111000100000001000000001000011000000000000000000000100010000000000001000000010100000  L-111_2 00000000000000000000100010000000111111111111111111111011100000000100111010100000000000000000010101000100000001111011111110001001111111111110101000000000000000001110000000111110010101111111111111111111011101111111111111101111110101100111111101110001101000011011111111100001101101011110011111110110011111101011111111111111111111110111111100000000000010010000000000101100110000100100111010011111101111111101011111111000010111111001110011111001000000000001101010011000000000000001000000000000010101100011111110111100100110010000011000000001000000101110011101010000100001001000111111111011101000001000000001111100100101111111111111111111111111100000000000011000000000001110001001111001111101001111101100100100001101100100011001100000001011000001001100010000000001111111111111001110000001111111100100001000000000001111101110110111111111111111111101110100000011011000110110110010111111111111001011011111011111000000010010110101100110011111111110001001110010111101  L-112_1 00000000000000000000001001111111001000000001000000100000100010111010000110100000000000000010011001000100000000000001100100001000000000000000101000000000000000000000000000000000000000011110100000000000000000010000000110000000000011100000000010100100000000000000000000000000000101001111111111110110010100100000001000000000010111000000100000000000000010000000000000000100110000100100000000000000000000000000110000000000000000000110001100011111101000000001000010001000000000000000000001000000010100100001111110111100110100010000111000000101000000001110011000110101011100100001111111111011101000001000000010111100111011100000000000000000000000000000000000010000000000001100000000010000100100000000000000001001101100011000000001100000001011000001001100010000001110000000000000101110000001111111000001010111100001001100001000000100000000000000000000001000000010010000100110100000110000000000001000000010010000000000000000000000000001111000000000011001000010000010  L-112_2 01000000001000000000101001111111001111111111111111100000100010111011000110100000000000000010011001000100000011111001111110001001111111111110101001100111111111111111111111111110111111011111111110100111101110010000000110000000000011100000000010100100000001000011111111011111100101001111111111110110010100100000001111111111111111000111100000000000000010000000000000000100110000100100000001011111101000001000111111111111111100000110001100111111101111111101101010001000000000000000000001000000010101100001111110111100110100010000111000000101000000001110011000110101011100100001111111111011101000001000000010111100111011100000000000000101000000000000000000010111100000001111010011011000101111111111000000001001101100011000000001100000001011000001001100010000001110000000000000101110000001111111100001010111100001001100001110010110000001000000000000001000111011111100110110110010110000001100011100000111010111110011110111111111110101111111111111111001000010011110  L-113_1 00000000000000000000101000001000001000111111111111010000000010111011000110100000000000000010011001000000000000000001111110001000000000000000000000000000000000000110000000000010000000011111110110011111001100000000000000000000000010000000000000000100000000000000100000000001100010100001111111110110010001111111111000000000000011110000111100000000000010000000000000101100110000100101000000000000000000001000010000000000000011110110001100011000000000001101101010010000000000000001000001000000010101100001111110000011011000010000000000000101000000101110000001000000011100101000011111111000001111110111110010111100100111100000000000000000000000000000000000010111100000001001010000100000101100000011000101101001101101000000011000100011101011000001001100010000001001111111111111101110111110010000000001011111100001001000100000000111000001000000000000000000000001011000100110000000110000000000001000000000010000000000000000000000000000010000000000000010100010100000  L-113_2 00000010011100001000101011111111011111111111111111010011100010111011000110100000000000000010011011100100000011111101111110001000000000000000010000100000000000001110000000100010111101111111111110011111001100011000000110000000000011100011110011100100000001010000111111001011100010100001111111110110011001111111111111111111111111111111111100000000000010010000000000101100110000100101000000001110100111111101110100111111110111110110001100111111101111111101101010011000000000000001000001000000010101100001111110000011011000010000000000000101000000101110000001000000011100101001011111111000001111110111110010111100100111100000000000000000000000000000000000010111100000001111011001100001111101000111000101101001101101000000011001100111101011000001001100010000011001111111111111101110111110010000000001011111100001001000101110110111111111111111111111110000110001111100110110100001111111111111011111011111010111111111111111111111111101111000000001101010110010110001  L-114_1 00000000000000000000100000000000010000100000010000000000000010111011000110100000000000000000010001000000000000000001101100000000000000000000000000000000000000001110000000100010010101011111100000000000100010010000000000000000000011100000000000000000000000000000111111100001100000000000000000000000000001000010000000010000000010110000100000000000000010000000000000000100110000100000000000001001000000000000010000000000000000001001110000011001000000000001100010000000000000000001000000000000010100000001100100110000000000000000000000000000000010001100001000110000000000000000001011111000000000000000010010111100100100000000000000000000000000000000000000011000000000000000000000010000100100000000000010000000001101000100000101100100000001000001001100010000000000111111111111101100000000010000000100001000000000000000001000010111111111100001011111110000000000000000001000000000000000111111001000000011010000000000000000000000000010010000000010000000000010100000  L-114_2 01000010010100001000101011111111011111111111111111101110100010111011000110100000000000000010011101100100001111111101111110001000000000000000001000000000000000001110000000111110010101011111111110011000100010010000000000000000000011100000000011100000000011000011111111100001101111111111100000000000000101111011111000010000010011110001110100000000000010010000000000000100110000100111001111011111111000001000111111111111111100001001110000011111101110111101101010011000000000000001000000010000010111101111100101110001111110110000111000000000001110101110011001110101111111100001111111111000001111011111110110111100100110000000000000000000000000000000000000011000000000000000000111010001101111111011000110101101101111000111001101100111111111000001001100010000000000111111111111101110010111111111100100001000000000011000101110010111111111111111111111110100111011110100111001010001111011111111001111010011010000111111111111111111111110011111111111101001110010110100  L-115_1 00000000000000000000000010000000000111111111111111000001100010111011000110100000000000000000010001000000000000000001101100001000000000000000000000000000000001110000000000000010000000011111100000011111001100000000000110000000000001000000000000000000000000000000111111000000000101010010000010001011100000000111111000000000010011000101100100000000000010000000000000000100110000100100000000011101100111111100010100100100100100000110001100000000000000000001101010010000000000000000000101000000010100100001100100111000110000000000011000000000000000000100000001000000000000000000000000000000000000000000000010000000100000000000000000000000000000000000000000010000000000001100000000000000101000001100000000000000001100000001010100000000000000000001001100010000000010100000000000101100000100000000000001010111100001011000000000000110000001000000000000001000000000000000000000100000111011111111001000000101010000000000000000000000000101110000000000000000000010011000  L-115_2 00111111101000000000101011111111101111111111111111110011100010111011000110100000000000000000011101000100000011111001111110001000000000000000001000000111111111111111111111100010111101111111111111111111011111111111111111101111110111100000000011110001100010011011111111111110001101011110011111111011101011111111111111111111111111111101111100011001000010010000000000101100110000100101001100011101111111111101110100100100100100000110001101111111101111111101101010010000000000000000011101000000010101100011111111111110111110010000111000001110000010101100100011110000111111100001000011111011101001111110000010111111111111111111111111111111111111100000000000010111100000001111011001110001111110111111000001101111101101111101111111100000111111100001001100010000000011111110111111101111111110010001011011111111111111111100001000011110000001000000000000001000000101111111111110100001111111111111011001011111010111000001010010110101100101111000000000001011110011111110  L-116_1 00000010000100000000001001111111000000000000000000000000000010111011000100100000000000000010010001000100000000000001100100000000000000000000000000000000000000000001111111000000000000010000000001100000000000000000000000000000000001000000000010100000000001000000111011000001100101011110011111110010011000100000000000000000010011000001111100000000000010000000000000101100110000100100111010001101101000001000000100100100100100001001110000000000000000000001100010000000000000000000000000000000010100100001111110110000100010010001000000000001000000000100000000110000000000000000000011111000000110000000010000011100000111100000000000000000000000000000000000000000000000000000000000010000001100001100000001001001100100000000000001000000001011000001001100010000000001110100000000001110000100010000010001010100000001101000001000000111000001000000000000000000110000000000000000000000110011000000000100000000010111000000000000000100000101110000000000000000000010110000  L-116_2 00000010010100000000101011111111001111111111111111001111000010111011000110100000000000000010011101000100000010000011111110001000000000000000011000100111111111110001111111111111101111011111111111111111111111111111111111101111110111000000000010101101100111000011111111101001111101011110011111111110011101111011111000111011111111110111111100000000000010010000000000101100110000100100111011011101101111111101111111100111101100001001110011011001000000000001101110011000000000000000000101001000010101100011111110111100110010010001101000101101000000001110011101110000111101110001011111111100001111011110010011011100100111111111111111111111111111100000000000011111100000000000000000010000101111111111000101101001101101100111111111100000101011000001001100010000000101111111111111101110010110010001011011010111111111111000101000110111111111111111111111110000110001111100101110100010111111111111001111010111010111000000000000001100000101111000000001111001110010111010  L-117_1 00000000000000000000001000000000001000000000000000000110100000000100111000100000000000000000010001000000000000000001100100000000000000000000001000000000000000000000000000000010010101011110100000000000000000000000000110000000000001100000000001000000000000000000000000000001100000000000000000000000000000010001111000010000000000111000100000000000000010000000000000000100110000100001000000000000000000000001000000000000000111111001110000000000000000000001101010000000000000000000000000000000010101100001100100111100100010010000101000000001000000000000000001110000001100101000001011111000001000000000000010011100100000000000000000000000000000000000000000010000000000000000000000000000101100000100000100001001100001000000000101000000001011000001001100010000000001000000000000001110000000000000010001010001000001001000001000010100000000000000000000001000000000000000100010000000000000000000011000000000010011000000000000000000000001110000000000001000110010110000  L-117_2 00100010000000000000001011110000011000000000000000000111100000000100111010100000000000000010011111000100000001111111111110001100000000000000001000000000000000001111111111000110010101111111101001111111111111111111111111101111110111100010111011010001101001000000100000001011100111111111100000001011100101111111111110010000000011111111111100000000000010000000000000101100110000100101000100011111111111111101110100111111110111111001110011011111101111111101101010011000000000000001000101000000010101100011111110111100110110010001111000000001000000111110011001110000011110101000111111111011101111111111110010011100111101100000000000110000000000000000000000010011100000001111011000100001111111111111000101001001101101100010110101111111111011000001001100010000001101110110000000101110000110010000011001110111111111111000101000010111111111111111111101111000001011011001110110000100100000000000011111011111010111111111111111111111111101111000000001011010110011110010  L-202_1 00000000000000000000000000000000000000000000000010000110100000000000000000100000000000000000011001000100000000000001111100000000000000000000000000000000000001110000000000100010010101111111111110011111001100000000000000000000000001100000000000000000000000000000000000000001000101010010000010001011101000000010000000000000010011000001110100000000000010000000000000101000110000100100000000000000000000000000010000001000010000000000000000011000000000000001100000000000000000000000000000000000010111000101100100000000010000010000000000000000000000000100000000110100000000000000001011111000000000001000000000100000111010000000000000000000000000000000000000000000000000000001011001100000101100000100000000001000101100000000000000000000000001000001000000010000000001000000000000001110000001111111100000000000000000001000001000010111111111111111111111110100000011000000110000000000110010000000001000000111010011000000000000000000000000011000000000001001000010100000  L-202_2 00000010001000001000101011111111111111111111111111110110100010111111111110100000000000000010011001000100000000000001111110001100000000000000101001000111111111111111111111100010010101111111111111111111011101111111111111101111110111100111110011110010011011000000111111100001101101011110000010001011101011000011111111111110111111111001111100000000000010010000000000101100110000100101111111001111101111111101111111111000010100001111111110111111101111111101101011011000010000000001000100000100010111101111100100111011110100010001001010000001000110001110011101110100111111101001111111111000001110001000000011111111111011000000000000000000000000000000000000010111100000001111011001100000111111111111000111001001101111100001010001111111001111000001001100010000000001111111111111101110000001111111100101011111100001011000101110110111111111111111111111110100111011111100110110110010111111111111001111011111010111111111111111111111111011111111111111101001000010111010  L-203_1 00000000000000000000000000000000010111111111111111100000000000000000000000100000000000000000011001100000000000000001111100000000000000000000101000000000000000000001011101000010010101011111100000000000000000000000000000000000000011000000000000000100000000000000100000000001001101001010011111110110010001101000000000000000000000110000100100000000000010000000000000000000110000100100000001010000000000000000110000000000000000001001110000011001000000000001100010000000000000000000000000000000010101100001111110000000101000010001001000000001000000000000001000000000011100101000011011111000000000000000000010000000011000000000000000000000000000000000000000010000000000000000000000000001101001000101000000000000001101000000001001100000000010000001000000010000000001000000000000001110000000010001000000000000000000001000001000010111110111100001111111110100011000000000000110000000110010000000000000000000010010000000000000000100000100011000000000000000000010100000  L-203_2 00000010010100001000001010000000111111111111111111100110100010111111111110100000000000000010011001100100000001111011111110001001111111111110101000000111111110000001111111000010010101111111100001111111101110011000000110000000000011100100000011100110000011000011111111101011101101011110011111110110010111111011100000111011111111111011111100000000000010000000000000000100110000100100111011011111101000001000111111111111111111111001110011111111101111111101101011001000000000000000000000001000010101100001111110111100111000010001101000000001000000101110011101110000011110101000111111111000001111011110011110111111111000000000000000000000000000000000000000011000000000001111010000110001111011111111000111101111101111100100111001100000101111100001001100010000000001000000000000101110000101111111110101011000000111111100101110010111111111111111111111110100111100011011111110000100111110001100001111011110010011000001000000000111100111111000000000001001110110111000  L-204_1 00000000000000000000001010000000001000000000000000000100000000000100111000100000000000000010011001000100000001100001000100001001111111111110000000000111111111110001111111000000000000011111100000000000000000010000000000000000000011000000000001000001100000000000000000000001100101011111100000000000000000100011100000000000010011000000110000000000000010010000000000000100110000100010001000011100100011111100000000010000000000001001110010011010000000000001100010010000000000000000000001000000010100100001111110111100111000010001001010000001000000101110011000110100011110101000111101111011101000001000000010011100100101100000000000000000000000000000000000010000000000000000000000000000111100000110100000000110001101000000010001000000001011000001001100010000000001000000000000001110000001111111100100001000000000001100001000000110000001000000000000000000000000000000001000010010000000000000000000000010010011000000000000000000000000010000000001000000110010100000  L-204_2 00100000000000000000001010000000001000000000000000100110100000000100111000100000000000000010011001000100000001111011111100001001111111111110000000000111111111110001111111110010111111011111111111100011101111110000000010000000000011000000000001010001100011001000100000000001100101011111100000000000000100100011100111111111111111110001110100000000000010010000011101101100110000100010001000011101101111111101110100011111111100001001110010011011000000000001101010010000000000000000000001000000010101100001111110111100111000010001001010000001000000101110011000110100011110101000111111111011101000001000000010011100100101111111111111111111111111100000000000011111100000001001011001110000111100000110100000000110001101000000010001000000001011000001001100010000000001000000000000001110000001111111100100001000000000001100001000110111111111100001111111110100110011111100001000110010111111111111011110010111011111111111111111111111111110011111111111110010110010110010  ;  End; |
| **NEX file of 158 SNPs from 20 muscle related genes** |
| #NEXUS  Begin data;  Dimensions ntax=46 nchar=158;  Format datatype=binary symbols="01" gap=-;  Matrix  H-101_1 10000000000000000010000000000000001010000000000000000000000000001010000100000100100000100111000010000000000000000000000000000000000000000001000000000000000000  H-101_2 10000000000000001111101100111110011110011101111010000000100111101010000100000100110000100111001111110100000000000000000000000011000011001001000000000000110100  H-103_1 10000000000000001000000000111000011011100000000000000000011110001000000100000000000000100111000000000100000000000000000000000000000000000001000000000000110000  H-103_2 11111111111111111100010000111000011111111011101011111100111111101010000100000100110000100111001000011100000000000000000000000010000001001111100000000000110101  H-104_1 10000000000000000000000000000000000100010010101001111100000000001010000100000000000000100111001000010100000000000000000000000010000000001001000000000000110001  H-104_2 10001111111111111110000000001111101110110010101111111101010100001010000100000000100000100111001011011100000000000000000000000010010000011111000000000000110101  H-105_1 10000000000000000100000000000000001100000101010001111100000000000000000100000000000000100111000110010000000000000000000000000000000000000001000000000000110001  H-105_2 11111111111111111111111111110000001110100101010011111110111110011100000100000000100000100111000110011000000000000000000000000010000001010111100000000000110101  H-118_1 00000000000000000001101111111000011100000101010000000000011110001000000100000000010000100111000010001000000000000000000000000010000000000001000000000000110100  H-118_2 10001000001100001111101111111000011110101101010011111111111111101010000100000100110000100111001110101100000000000000000000000011000001101111100000000000110101  H-119_1 10000000000000000100000000001000001000000101000010000011111111101010000100000000010000100111000000000000000000000000000000000000000000001001100000000000110100  H-119_2 10000000000000000110010001001000011110111101010011111111111111101010000100000000110000100111001101001100000000000000000000000011000000011101100000000000110100  H-120_1 10000000000000001110000000001000001000000101010000000011011111001010000100000000010000100111000000000000000000000000000000000000000001100011000000000000100000  H-120_2 10000000000000001111101111111000011100010101010011110111111111101010000100000101110000100111001101111100000000000000000000000011000001101011000000000000110101  H-121_1 10000000000000000110000000000000000010000000000010100000111110001000000100000000000000100111000110000000000000000000000000000000000000000001000000000000000100  H-121_2 10000000000000001110000010111000010110011111111011110100111111101010000100000000110000100111000111011100000000000000000000000000010011100001000000000000110101  H-122_1 10000000000000001011101111000000010100000101010000000000000000001110000100000000100000100111000001000000000000000000000000000000000000000001000000000000110000  H-122_2 10000000000000001111101111111000011110001101010010010000000000001110000100000000110000100111001111011100000000000000000000000001000000001111100000000000110100  H-123_1 10000000000000000000010000001000000010000100010010000000000000001010000000000000000000100111000000000000000000000000000000000010000000000001000000000000110000  H-123_2 10000000000000001100010000111000011011101101010011111111111111101010001110000001100000100111001110111000000000000000000000000010000001111001000000000000110101  H-201_1 10000000000000000110010000000000000000000000000000000000000000000010000100000000000000100111000110001000000000000000000000000000000000000101000000000000000100  H-201_2 10000000000000001110010000000000001101111111111001110100100011111110000100000000000000100111000110001100000000000000000000000011000001011111100000000000110101  L-108_1 10000000000000000100010000000111100110100000000001111100000000000100000000000000110000100111000110001011111111111011111111111110000001110001111000000000000000  L-108_2 10000000001000000100010000000111101111110111101111111100000010011110111111000101110000100111000110101011111111111111111111111110000011110001111100000000110000  L-110_1 01111111111111110100010000000000000110110010101011111100000000010100000100000000110000000000000010000000000000000000000000000010000001010001100011111110000000  L-110_2 01111111111111111100010000000101001110110010101111111100000000010100000100000101111111111111111110011111111111111111111111111111010011110001111111111111110100  L-111_1 11111000000000000100010000000000000010010010101011110100000000001000000000000100111111100111000110101000000000000110000000000011010011000001000000000000000000  L-111_2 11111111111111110100010000000111101111110010101111111100111111101111111111111111111111100111000110101011111111111111111111111111010011111011100011111111001101  L-112_1 00000000010000000100010000000000000110000000000011110100000000010100000000000101110000011000110100001000000000000000000000000001000000000000111100000000000010  L-112_2 01111111111111110100010000001111100111110111111011111100000000010101111111111111110000011000110110101000000000000000101000000011111111110000111111111111111110  L-113_1 00001111111111110100000000000000000111110000000011111100000000000000000000000000111111011000110110101000000000000000000000000011000000110001100000000000000000  L-113_2 11111111111111111110010000001111110111110111101111111100011110011101111111111111111111011000110110101000000000000000000000000011010001110001111100000000110001  L-114_1 00001000000100000100000000000000000110110010101011111100000000000000000100000000100000100111000110001000000000000000000000000001000000000000000000000001000000  L-114_2 11111111111111110110010000111111110111110010101011111100000000011100000100000100110000100111000110101000000000000000000000000011111110110001100011111111110100  L-115_1 11111111111111110100000000000000000110110000000011111100000000000000000000000100110000011000110110101000000000000000000000000010000011000000100000000000001000  L-115_2 11111111111111110100010000001111100111110111101111111100000000011111111111111111110000011000110110101011111111111111111111111111101111110000100100000000001110  L-116_1 00000000000000000100010000000000000110000000000010000000000000010100000000000100110000100111000110001000000000000000000000000011000011000000000000000000000000  L-116_2 11111111111111110100010000001000001111111101111011111100000000010100001110111111110000100111000110111011111111111111111111111111111111110001111000000000111010  L-117_1 00000000000000000100000000000000000110010010101011110100000000001000000100000000001111100111000110101000000000000000000000000011000001000001000000000000000000  L-117_2 10000000000000001100010000000111111111110010101111111100010111011011100100000000111111100111000110101000000000000110000000000011111111110001000000000000100010  L-202_1 00000000000000100100010000000000000111110010101111111100000000000000000000000100110000000000000110000000000000000000000000000011000001000000100000000000000000  L-202_2 11111111111111110100010000000000000111110010101111111100111110011111111111101111110000111111110110101100000000000000000000000011111111110001100011111111111010  L-203_1 01111111111111110110000000000000000111110010101011111100000000000000000000000000000000100111000110001000000000000000000000000010010001010000100000000000000000  L-203_2 11111111111111110110010000000111101111110010101111111100100000011100001110111111111111100111000110101100000000000000000000000010111111110001111100000000001000  L-204_1 10000000000000000100010000000110000100000000000011111100000000001000000000000100110000100111000110001000000000000000000000000011000001101000100000000000100000  L-204_2 10000000000000000100010000000111101111110111111011111100000000001011111111111111110000100111000110101011111111111111111111111111000001101000100011111111110010  ;  End; |
| **NEX file of 956 SNPs randomly selected from genome** |
| #NEXUS  Begin data;  Dimensions ntax=46 nchar=956;  Format datatype=binary symbols="01" gap=-;  Matrix  H-101_1 00000000000011000000000000100000100000000100000000000000000000000100000110100000000000000000100010011000001001000001000000011000000011000000000000000000100100010000001110000000000000000000000000001000010000100000000000000110100110000110000001000000000001000000000100100100000010100010110000001001000000000000100000000000000010000010010000001000100000000010100000001000000100000000100000000000000100010000111010000001100000100010000000001000010000001000001000100000000010000000100000100001000000001001000001000000000000100000001101100001100001000000001000000100001000000000001000100000000000000000100000000000000001111000000000010001100000000110000100000100001110000000011000001000010101000000000100000000001100000000000001000001110001110000000000000000000001000000011101110000100000000101000000001000000000000100000000000000000000000100010000100000000010100111010000000000001000000000000000000000101000000000010001101100010000001000010010000100000000000000  H-101_2 11011010011111110011000001100010111000010111110010001010001001000100010110100001010111100001111110111011111101111101010010111011110111110100000000001001111110111011001111001001110010011100101100011110110001110100101000110111100111100110111101000000010101111100000100100110101010101111111101011101010000111101111100001100110011100010011100101001101100100011100000001110010110000010110101010000101100011101111110000001110000100111010010011100110010101001001110100001101010000110100001110111100010001011000101010010000000110000111101100111110001001110001111100100011100011001101100111100001101101111101000100000010011111110001000110001100010111110100110001100001111011010111000011011111111011011110101100100101110010110010001100001111001111001000010000011100101111101111101110110110101110101110100111010001100001100101001010100001000011100111010101110011010111111111101001000001001000011100011001100101010100000111011111111011111001101011011111110101001011110  H-103_1 00001000000110000000000000000000100000000000010010000000000001000000000100000000000000100000000000000001111011000001000010010100100010000000000000000000000110100000001100010000100100000100000000001000000000000100000000000010100010000010101000000010000000000100000010000010000000100001100100001001000000001000100100001000100011100000000000101000000000100011100000000010000100100010100100000000000100010100010110000000101000000100000000000100001000000000000000010000000000001000000000001101000000001011000010000010000000100000000101000000100000000011000100100000001000100000100100101100000000010000100100000001010011000000000000000000000010010000100000000000001110000000001000000000010011010100000110000000001001000001000001000100000010100000001010000010000000100000000001110000110000100001000000000000000000000100000000010000011000000000000000011010000000000000000000010000011000000010000001000001001001000010010000101101010000000100000010010010000001010000  H-103_2 10001110000111111000001000100110101000010110110110011100001001001110110100101001100110101010011011111111111011001001110110110100101011100100001111110000100111100100011111011100101111000110101010101001010010100110001100011110111110111110111001101011011001110101010110000010011111101011111101001101100100111011100110001000110011101000011000101011101000110111111101001111000111100111101110001100000101011100111110000011111010110110011001111101011110010110001101111101010010101101100000111101101010001011001111000010000000110000011111110100110011001011001111100110111011100001100100111100011010011011111101000101010011111100111000100100100010010110101100001001001111000000011010111001111011110100100110110100001011001001011111000100010110110100001010111010101101101000011101111001111101111001101110000101111111011100110011011111111011011101111111111011001000100010110001010000011000000011111001001001101101111111110001111111111100001111110010011110100001010001  H-104_1 00001000000010000000000010100010100000000000010000010100001101000110000010110001000000100000000000010001010001000001100001000000000011100000010000100000100100101001001101010000000000000100100000000000000000100000000000000110100001000110011000010010011001010000000000000001100110100011000100000000000100000000100100000010100000100100011000001000011001100011011100000010010100000000000100000001000100000100111010000000101010000100000000000000000000001010100011001000001000000000100000001000100000000001000001000000000000000000011101010110000010010000001010000010000001000001101100101000000000000010101100010100000001111001000000000000000000000010000100000101001110000000010000011000000001000000000010100000001000010000001001000001111000101000000000001000100000001010010001101000000101000000000000001000000101000000010101011000000000010010000000001000000000100001110100000000001100010010000001001000100001001000010001100000001000101100110010010100000000111000  H-104_2 00001000100110010000001110100010111010000101110001110110011111101110100010110101000101110001010110010001111111001001111001000001001011100001011000101011101110101111101111010010110100000100111000101011100000101000000101110111100101100110111001111011111001011110001100000111111110101011111101111001101110111011100100001111101111100100011000111011111001100111111100010011011110110010110100011001010101011101111010100010111010110101100000000000000011001010101111101000011000100110110000111111100100111011010101000011100000000000111101010111100111011001001110011110101001010101101110111100001000100111101110010111100011111001000000100001101000110110000101110101011110000010011001011101110011000001010111110100111111010011001111100101111100111001101010001011100001101011110111111010010101011011100100111000010101011100110101011101111111010110111001111100000010100111111101101111111111011011110001011100101001001010110001111111011000111111110011011110111011111001  H-105_1 00000000000011000000000001100010011000100000000000001100000000001010000110100010000000100000000000000000001001000101100000000010100011000000000010100000100110001000101111000000000100000000000100000010000000000000000000000100111000000100100010000010000000010000000101000010000000000000001000001001001000010000000000000000110000100100010000000000001000100001100000000010000000000001100100000000000000000100001010000001101000000010000001001000000010000000000001000000000010101000000000100000000000001000000000000000000000100000000001000100000000000001000100100000001000000001000000011100000000000100100000000000010000001000000000000001000000000010000100000101001100000000000000000000000101000000000100100100011010000000000001000000000000000000000000000010100000001000000101110000000000110000000001000000010000001100000000010000000000000100000100001010000000000010000001110000011000000011100000000000001000000000010000110001010000000000000000000100000000011000  H-105_2 01101011010111100100000101101010111000101111110010101100000011001010000110100110011101110010111111001111111011000111110000010010100111000100010010110000100111101011101111011101010100000100011100001110010000100110001110001111111001111110101011001010110000010101000101010111011010100011111101001001101011011001110100001000111111100110011000001000001010100011100010000110000101100011101110100011101100101101011110011001111000110111000111001000011010010000011001110100001110101010010011101000011001101111011111110100000010110010011101100110000000001001001100110101111100010001011100111100100000101111101011000001011000111001101101110011100010110010011100101101101110010001011101100000011101000010101110100100011010011001010111000011110010111001101010000111110011111001011111110011010101111111100101100010010110001111100101110101001011011100011110101010100000100010110001110110111010110011101000101000101101001110011100111111011101001111010111011110010001011001  H-118_1 00000000000111000001000100000010110000000000010000000000000111000000010100100100000000000000001010010001010001001001000000000000100001000100000100000000000000001000001111000100000100010000100000001010000010000000000000000100100111010110110001001000000000110100000100000010000000100010100000001001000000001000100000000000100001000000000100000000011000100011110000000000000000000000000100000000000100010110001010000000001000110000000000010000000000000010000000000000000000000000100000100001000000000000000100000001000000110000010111000010100000000010001100000100001000000000000000101100000000000001100011000001011001101000001100100001000000000000000000101100001110000000001000010000000000000000100100000000001000101000000001000000000100111000000000000010100101100000011001110000010000010001000100000001000110101100000000000100000000000100000000001000001010000010010000000000000000000000000000000001000000000000000001101101000100001000010010001000011001000000  H-118_2 11001110100111101001000100101010110011010000010001001011100111001000110110100100000101111000111110111011011101001011110100011011111011100101010100100100100001101000101111110100000100010100111000001010000111000110010110000100100111110110110111001000111001110100100101111010011011110111110001001101010001011000101100011111110111001011011100111000111111101111110010000010000101000111111111001001000101110111011110011000111000110110000001010000010010001011000101110000000010001111100010111001101110101101101110000011011100110110011111101110101101000110001110000110111000100011111100101100001010001111100011000101111101101110001101101111111001010101000110111100101110100000011011011001111111011101100110110100101001111101000111010010100110111010001000000011111101100101011001110000010101010101100110100011001110101111000001010100001010110110010101101010001011100011011111010001011110011011100001100001100011001100000001101101011101001111010010011110011101011011  H-119_1 00000000000010000000000011000000000000000000010000001000000000000000000000000010000100101000100011000000000000000001000000001000000001101000000000010000000110001010000110010000010100000000100000000000000000100000000000000111000110000010010000001010000000010000010000000010000010000000000000001001000000110000100000001000000001100100001000000000000000000001000000000010000110000000100100000000000100000100000110000000001000000100010000000000000010000000001100110100001010000100000000100001000000000001100001000000000000110010010100100000000001100001000010100000000000000001000000000100001100000000101100000001010001100000000000100001000010000010000000000100001100000000000000001001010000100000000010100000000000010000000011000000000000000000000000000010000001100000011001100000010001000000000000000000000000000100000000010100000000000000000000000000000000000010000000000000001000000010100000000000000000100000010000110111011100001010010010001000000001000000  H-119_2 01011000000111100101001011100010111010000000010110011100011001110110100101110011100101111000111111011001011011000101010011101110000011101101010100110111100110001011011110010110110110000111100011000010010100100100011000000111100110000010111100001010010001010100010001011110111110100111101110011101010100111100111100011000110011101111001001001000101000101011101100001110001110110010100110111001010100010101111110001010101011011110010001011111000010101101001101111100001110100110000000100001101011011001111111001000100010110010011111111111111111100001000110110111101001000101110000101100001110100111101110001101010001111000001001110011100010000010000100110110111110010000011001101001010101111100101111110100001000110011001011000100100100110001000000101010000101110100011101111010111111010011110100101010010000101100110001011110001111000101111010001110001010100111111101000100011000110111111101110001111000100111010100111111011100011011110111111110100001111000  H-120_1 00001000000010000000000000100010010000000000010000001100000100000100000100000101000000100000000000000000000001000100010000001010001000010000000000000000100110001000001111000000100101000000100000000000000000100100000000100001000111000110101000000000000011000000000100000010010010100000010101001001000000100000000000000000100011100100000000001000001000100010000100000000000100000000000100000001100000000100010110000011100000000100000001000000000010000000000100010000000010000000000000001001000000000000000100100000000000110010011101000000100000000011000100000100000000000000101000101100000100000100100100000001110000100000000000100001100010000000000110000110000010000000000000000000000000110000000000000100001000000001000000000000000100010100001010000000000001100000010101000000001101010001000000000000000110001100000000010000000001000000000100001110000010100010010000000000011000000011000000000000000000000000010000010001000100000000000000010000000001011000  H-120_2 00001000010111000011010001100010111000010101110001011100011111001111111100101101001100111010101111011101111111011111011001001011111011010001111000000010100110101010001111011000101101100100101000111001010001110100111100110001100111010110101001001000110111010100010111010010010010101001110101001101001000111001100100000100111011110110101011111001001000100010100100100110011110000000111110010001100101010100111110010011101000110111111011001101110111111000001100010100111011101100000000101101111111101000010101100000100100110010011101100101100000000011001101100110001100100101101100111100100111001110101100000011111001111000001010100001101110010110001111100110111110000001011001101001110011110001110110111101011011011011100101000010110100010100101010000011000101101100010111111010111111011001101010010100000110001110000000110100000011000100011100001110100011110011011101010000011010000011101011000000101000000111010010111001110101001110001011011100100001011011  H-121_1 00000000000000000000000001100100000100010100000000000000000001010000000000100000010000110000001000010001001001000001010000000001000010000000000000110100100000001000011100000000000000010000000000001000000000100000001000000010100110000100101000000010000000010100000000001011000000100101101000001100000000010000000000001000110000100100000000100000000000100001000000000000000100000011100000000000100100010100101010000001001000000000000000000000010010000000000000000010000010000000000000000100100000001001000000000000000010000000110001100000100000000000001110100000001000000001101000100110000000010001101000000000011000100000000000100000000000000000000000000000000100000000010000011000010101000000100000100100100000000000000001010000000110000000001001010000100000000000010000100000100101000001000100000001000000001100100000010000000010000000000010101000000010000011000000000000001000000000100000000001001000000010000001011000010100000100010010011000000000010000  H-121_2 10001100000110001010001101110110101110010101110000000100000011110110000000100010110101110011011110110001111111010111110100100001111111110101011000111100100100101011011111011110000100010110111001001110000011111110001100100011110111110110101000001011100101011111110110001111001010101101101101001111000000011101100101001001110011100110011000101000101000110001100010000010000100100111110110010100101100111110101010001101111000100110010000010100010110000001011011110111010010000010100000110110110000001001101101000011000110100001110111100101110001001001101110110111101000001001111110101111001000110111101000101011011011101000011011100101100011100011000101101000000110110110011001111001010101000100111110101101101011011001000111010101111111110010101111110001111001111000011001110011110101011011100111100001010000101110100000110100010010011000111110101101000110010011010101000101001000001110100101100001101110010110111001111111111100001111010110111101000001010011  H-122_1 00001000000010000000000000000101100000000100010000010100001001000100000100010000000100000000000010010000000000010010000000100000000100000000000010010000000000011000010110000000000000010000010000000010000000100100000000000000000110001000111000000010100001110000000001000010000101100000001101001001000100011010100000000001100000000100010100000000000000100011001100000000000110000000100100000000000110010100011110000010101010000100000000001000010010000000000000101000001000100000000000100000010000000001000101000000000000111000000100010101000000010000000010000100001101000000100000000100000000010001101100000001010001010000000000000001000000010010000000001000100110000000010000010001010000000000000010100100001000110010001001000001100100010000000010001000000001100000010001100000100100000001000000000000010101001100010001011000000011011000110000000000010100000000000000000000001000000001010001000000000010100000010000101100000000100000110000011100000001111000  H-122_2 10001001001011100111011101000111111011000110110111010101101001011100011110110010010111111001100110010100101001010110001011101011000111011111111011010100101110111010011111110000010000010100111010101110010001100110001100001011100111011110111001001010100111110111010011010010001111101111001111011101001100011110110110101001101111100100111100110011100100110111001100000110010110110011110110100010000110011100111110001010101010111110010100011100010111000100001101101100001011101110101100100101110000011011000101011100000100111101111101010111110011010001101110000110101101000110101000101100001000110111101100101001011001011000111000100101100000111011100110101011101110001000011001011101010000000100110010100100101011110010001011000111110111110001010110001010011011111010011101110010100101010101100001110000011111111111110011011101001011011100111010011010110110111111011001010101001010000001110001100100101011100010111010101111011000111110110010111110101011111001  H-123_1 00000000000011010000100000000000000000000000100100010000000001000110000100010101000100100000000010000001101011000000000000000010000011000000000010010000000001001000000110010000000000000000001100000000000000000001000000000101101000000100100000001010000000010000000000000000000110000000110000000101000000000010100000000000000000100000001000100000000000100011001100000010010100010000100100000000000010000100010110000001110010100110000000001001000010000000000001001000000100101010100000000101000000000001000101000010000001100010000101010000000101000010001110000000000001000100001001101100000000000001100001000000001000101100001001000000101000001010000100000000001010000000011000000000010001100000000100000000000000000001001101000000100000000000001010001100000001110000000101101000110001000001000000000000000001001100010000001000001000000100000100000000001000100011000000001000001001000010110001000000100000000011010000110101001000011000100010000100000000100001  H-123_2 00001011100111010000101000110010110010010011110100011100101001000111001110010101000100101011110010001011111111001111111001000010011111000101101010110100100111101110111111010111110001100110111101001110000000000001000010000111101100000110101101011010000111010100011110000010010110100111111110001101001100010011100110001010101010110010011000101111100000111011101100000110010110010001101100011011100111010100111110000001111010101110000010101101001011000000010001011100010100101110111111100101110001011001000111000110001011100010100101110000101101000111011110110010111001100100101101111101001001000111100101001111011001101101001011100011101100111010100100100111101110010100011010001011111011101100101100110101001100010011011101000111111111110000011111011110000001111110010101101011111001000101110101010110001101001110010011001100011100111101011111111010011010111011011101101010101001001011110001010101101100000111011001111111011000011111110010010111001100111001  H-201_1 00001000000100100000000000100010010000000100100000000000000000000100000110000110100000110000001000011001111000001100000000001011001011010000000000011000010000001000011111010000000000000000000000100000000000110101000000000110100000000010110000001000001100010100000000000010000010100001001001001001000000010000100000001000000011101000000000000000010000000100100000000000000110000000100100000001000000010000000010000001000000110110000000011000010010000000001000010000100000100010000000000000100000001000000001000000000000011010001101000000100000000000000000000000101000000000000000101000001000000100101100010001011001100000000000100011000010000010000100000000101111010000000000000011010001000010000010000001000000001010000000000001110100100000000111001000100000010000010001001000000101010001000000000000000000101000000000010000001011010000011010000000000000000010010000100100011000000011001010000010001000000000000011000101000001000100010010000100000001010100  H-201_2 00001000010101110000011001101010111000110100110101011100001000101110101110110111101001111000001100011101111010101101010100001011101111010101010000011000110100101110111111010101000000001100101000100110111100110101011100010111101001001110111000001000111111010100000100000110001111100011111001011001000101111000100100001000101111101000100000001100111000100111101101010010000110010110110100000001101100010100101111100011011010110111110001011000010111001000001001111000101110101010000000000001111111001001000011000000000010111110111101010011100111000001001001100110101001000011101100111100001010111100111100011101111011110000111000100011010010110110001101000010101111010000011011101011110101010010000111100101001100001011101111000011110100100000000111011010100001111001010101101010100111010001100110100010001100101110010000111100111011111100011010011000010010110011011101101100011000111011111010101110101101001110000011011101000001001111110010010111000001111101  L-108_1 00001000000000000000000001000000100000000000000000000000000001001000000100000111000000100000000010010000001000001000000000000000000011010010001000010000100100001010001101011000000000010000100000001000110000100100000000000110100110000010011000001010011001010100010000000010000000010001100100001001000000000000100100001000110000100000010000100000000100100011010000000000001100000000110000000000000100000000010010000000100000000100000000000001010010010000000001000000000100000100100000000001100000001011000001000000000000110000000000000000100000000000001000000000000000000100001000100110000000000101001001010001000001001000000000100001100000000010000101001000001111000000001000000000110001000010100110100000001100010001000001001001000000110001000000000000110100000000000000100000000100000001001010000000010100001110000000010101001010000100010000001000000010000000001000000000001000000010000000000000100000100000000000010101000010001000010010000100000000010100  L-108_2 00001010001011011011000101111010111110011001011010111110000001101110000110100111011011100110110010010011111011011011110100001000011011111011001000010100110110111011001111111110110011011101101000001010110100100100001000000110110111100110111001001111111001011100111001001110011110111111110110011001001000011000110101101000110011100011111000100010101100110011010000110011011110000011110100010001010101011110010010011010101001111101001010110001110111010001000101010000000101111111100000110001110110001111011101001101000010110010111001000001100100000110001001010100111100001100001000101111000000000111101101010011111101011100001011110011101000100111101101101000101111100111011010011101111111000110100111100111111110010101100001001001110101110001001110000011111111110011000101111010111101010101101110100111011100011110000001010101001011010100011110101010001010110011111011010001011000001011100000000100101011101111110110111101011011001111010010110101101001011100  L-110_1 00000000000011000000000001000100110000000001000000010010001000000100000110110000000100001000010000000000001000000001000000000000000001000000000000000000000100001000001101011000000100000100100000001000010000100000000000000000100000000101100000001010010001000100000001000010000110100010001100000001000000000010100100000010000000100000010000100000000000110001001100000110000100010001100000000001000010000100110010000000000010100111000000001001000010000000000001001100010000110000000001000001010000001000000000000000000000000000010101010000100100000000001000001100001101000000001010101100000000000101100000000001000001100000001000100000100000011010000000000100000110000000011000000001000001000000000000100000000010001001011011100001000100000000001010001010000000100000011000100000100100100001000100001000000001000110010000011000000010000000000000100000000000100010010001000000010000000000011000000000000000000000000001101101000000000000110010010100000000110000  L-110_2 01111000010111011001001101110110110101011001110000111011011111001110000111111010010110101010010110010001011001001101110001011001010111000100001100110001100110101110001111011000110100000100101000011011010001100000011010110000111101101111101100011011011001010100010101010110100110100011001100100101101000101011100100010110110110100001011001100010111010110111101101000110000110010011100110001001000110000101111110010011001010110111110000001101010010000101100111011110010110110010100001110101010000001001001111110001001010111000111111011101101111000000001100001100101101000010101011101110001000000111101101110111010011110100001001111001111111111111000100101101001111000000011111011001110001010000110100100100101110011001111011111001100100110000101010101011100101110010011101101110110101110001101100101010000111001110111010011100000010010110011110101010000010100011011011010010011001000011111110010100111110100110100111111111011101011100111011011101000001111011  L-111_1 00000000000001000000000000100100100000000000000000000100000001000000000100000100000000000000000000001001001001000001100000000000000011000000000000000000100110000010001110000010010000000000101100000000000000100001000000000110100011000100010000000000000001110100000001000001000010100011100101000001001000001000100100001000100000100000001000000000000000000001000000000010000100000000100100000000000000010100010110000001001001100000000000001000010110001100100000100000000000000010000100000010101000001000000000000010100000100000011101000100100000000000000000110000101010000000000000101100000000000100101000000100000000101000000000100000000000110000010000000001001010000000011000000000010100000000100110100000000000010010000001000000000000001000001000000000000000000000010001110000000100000001100001100000000100001100000001010000000000000000000000010100000010010011001001000000001000000010100001000000001000000000000001101100011110001001010000000010000010001000  L-111_2 00011010000101101100001101100100101100110100110011001100000001101010010111100110001001111011100101011111011001111111110000001101001011000101010000100000100110101010001111011110111100000110101100101010000001100101001100001111110111110110011110101100110011110100000111010111010010110111111101001101101001101100110100011101111010100010011000111011001011100111100000110110100110000001100100100001101000111101011111000011101001110110100000111101111111001100100011100100011100010111100101010111101000001101000100011111111001110110011111000101110101100100001000110100111110001001001000101100101000100101101100000101010011111010000001110011100010110111110100011111101111010001011011001001111111001011100111100000001001010011000111100001010111111000101100000011000101110100111001111111100110000001100101110111000100001110001001010100001011011100111000111110101010110011011001110010101010000010101001101100101100010111000011111111011110001111010110111110001011011001  L-112_1 00000000000101000000100000000110101000000000000100111100001000000000010100110101010000100000110000000001100001000001000000000000000001000000000010100000000110001000000111010000110100010000100000000000000100100000000000000001100000000010101000001000000000010000000000000010000110100010101100000100000100001000100000000000101010100000001100000100000000100011001100100010000100010000100000100000100110000100010100000001000010000000000000000001001010000100001000001000010000001000000000000000000000001001000001000000000000010000010101010100000000010010000010000000100101000000000000101000000000000000000100000001011000101000000000100000100000100010000100000000001110000000001101011000000001000000100010100000000000000000001001000000100000001000100000001000000000100000010001010000000100000101000000000000000000000010010100011000000000000000000100110010000000100011010100000000000000000000011000000000100000100010000010111001000100011100100010000100000000100000  L-112_2 00001000000111111100101000110110111000010011001100111101001011000100010110110101110100100011111110111001111101000101110100000010100111101101001110100100100110111110001111011010111100110101100001010110010111100110001100100011100100000110101011001001000011010100100111000010000110100011111111101111101111111110100111000110111011101000011110110110001011100011001110100110010100010000100110110100101110110100111111100011001110000110010101010011001111000100001000001000011010001100111100000100101110001101001001000011011111110010111111010101100101010011111110011110101101100010100100101100001000101101100100000001011010101100111001110011110111111010101101000100001111001010111101011111110011000000100010111100110001110001011111100011110010101101111000001010001000111110011001111010110101011111100000010010100111101111110101111110010111011101111110111111110100101011010101110110011101011110111101000000100100100111000010111001111100111111100011010100100000111001  L-113_1 10000000000010100000000000100100010000000000000000000110000000000000000100000100000000000010000000010001010000000001100000000001100010000100000000000000100100000010001111001000100100010000101000000010000000100000010010000100000100000100101000001000010000000100000000000000000010000011101001001101000000000000000000000000100010100000010000000010000000100001000000000110000100000000100000000000000000000000011110000010111000010000000000000000000000000000000001000010010000000000000000000000010000001011000001000000000000100010000000000000100100000001000100000110001000000000000000101000000000000000000000000001000100100000001000000000000000000010000100000100001110000000010100011001010000000000100000100000001000000000000001000100000000000000100000000010101000110000010001110000100101001001000000000000100100001100000000110000001100000100000000000010000000100010001000000000001000000010000000000010100000100000000011011001001010000000010010101000000000001000  L-113_2 10110000000011111000010101100100110000000100010000001111100001000100010110100101000101111111011110011001011101101101101000101001101011000111001001100110101100101111001111101000110100011101101000000011001101111100010010000111100101010110101011011100010111011110010111011110011010110011101001111111011000111001100100100010100011110000011000101010001100100111000010000110010111000011110100000011101101000100111110001010111100110110110001010100111010101000011101010010011010001110110001110101110110001111000011010010000000110010101100000101100101100101001100100110001110001000101100111101101000100110101100000001110101111000111000110010110000110110100111100100111111010011010101111011010101010000100100111111101000001111000001000101100111110011101010000010111111110010010101110111111101111111100101100111101110001110001000110110001100110100001000010010000110110011011111101011111100100010101001010010111011101100010011011101011010001001010011111100100001011000  L-114_1 10000000000010110000000001110000000000000000010010000000000000000000000110000001000000100010000000001001000001000101000000000000100011000000010000000000100100001000001110001000100100000000001000000010000000000000010000000111000001000010010000001000000001100000000100000010000010100011000100001000000010010000000000001000101011100010001000000000001000100000000000000010000110000000100100000000100000000000001000000001100000010000000000000000000010000000000000000000100000100000100000100100000000001000000000010000000000100000000101000100000000000000000000000000001000000000100000101100000000000010000110000000000000000000000000000000100000010000100000000001001100000000110100001001010000000000100010100000001000010000000001000000100000010000000000000001100000100000010001100000000000000000000100101000000100001100000000000100000011000000001000000000000000100010010100000010001000000010100000001000000000010010010000100001010000001100000000000100000000000000  L-114_2 10010000100011110001001001110101010000000001111010001100100001000010000110100111000101110011010010011001011101001101110001000011110011000000110001100001101110111011001111011111110111100010111010100010011000101111011100100111100111100010111010011010000011110110100100010010100010100111110110011001001010111000110011001011101111101111011100111000101000111001100001000011000110001011101111001111100101010101111001000011101000110111110010000011010010001101000100110000110001110010110011100101100000001011101111110010001010111111011101101101110001010110000110100101101100000000100101111101011000000111110111000011010001111000001010100100110010110011110110000111101111110010111101001111110111101110101110101111001100110001010001100001100000110001001000100101100101101000111101111011100101111001100111101001001110001100100000010100110111010100111110101101010110110110010101010111011000101111101101101000100001010011010011111111011001001111011011010101110001001001  L-115_1 00001000000010110000000000100000001000000000000000000000000001000010000100100000000000101100000110000000001100001001000001000000100001000000000000000000100110000010001010010000010000000100100000001010000000110000000000000010100000000100001000001000001001000100000000000010000010100010101001011001000000010000100000000001100000100000010000000010000000000001000000000000000000000000110100000001000000000100110010000010001000100010000000010001000010000000000001000100000000000000000000000001010000001000001010000000000010110000000100000001100000000010000100000000001000000000000000001100001000000100101100010000001000000000000000100000100000111010000100000100101000000000111000000001000000000000100010100000000000000000000000000000100000110000100000000001110110000000000101100000010000010001000100000000000100001100000000100100001000000000010000100000000000100011000000000000001000000010001001000000000000001000000000000101011000001110010000000000000000010000  L-115_2 01101011101111110101011101100010101001010000100000011100101001001010001110100101011111101110010111011011011111001001100001100001111111010000011010010000110110101110001111011000010000110101101101111011000000110000101011100110110110100111111010001110001011110101000111000010010110100011111101011101100110011001100101100001100010100010011000011010101101100111001110000110001110110000110101000011100111010100111110100111111010111110101001110011010111000001001101011111001010001010100101100111111011101101001011010001001010110111010111111011100100000011111100100100111011000001001001111101101000010111101111010001011111110001001000110010100000111111110100000100111110100100111001111011011110110000110111100110000001010011101011000001100101110111111000011111111110110011000101101010110101010001101100000110100111001100111001111110001011010100111101111010000010110011010101011000011011000111101011100101101000101111110110110111011101011110110110011100101101011110  L-116_1 00000000000011100000000001100100001000000001000000000000000001000010000110100000000000101100000000000000010100000001000000001000101011000000000000100000000000001000001110000100100000000100100000010001000001100000001000000010000000000100010000001000000000010000000000000010000000000001101100001011000000001000100000000000000011100000010100000000000000100010000000000110000010000000000100000001000000000100011010000000101000010000100000001000000000000000001010000000001100010000100000001001110000000000000011000000000000100000011001000100000001000000001100000100000000000000100100010000000000000010101000000000010001000000001000100001100000010011000100000000001110000000010000001000010001000000101010100000000000010000000001000000100000011000001000000000100000100000010000100000010100000001000000100000000000001000000000010100000000000000000000100000000100110000000101000000001000000010100001000001101010000010000000111110001000001010000010010000000010000000  L-116_2 10001101000111110111011011110111111011110001100000011101010011001110000110101101000101101110110100011001011100011101111110001001111011110000010010100110100110101000001111011100100100010110100000011011011111111101001000100111110111000110111110001000010101110110100011001010010011100111101101111111001000011001100010000000100111100100010100011010001001100011100000111110000110101110100101000011110100010111011010000111101001110100111000011101010110100000001011000100001110111111100001101101110000101001010111010000010000110010011101100101110101100001111100110110101110000011101110111110011001100111101001110100011001111101001000110001110010111011111100000101101111011000011001011000010101101100111110100100001001010011110001100101100101111000001010000001100000110011010101111111010111011001101010100010011110011101100001110111100000111101111110110011010111110110011101000001011000010111101001000101111110101111011001111111011100001110010010111100001011010101  L-117_1 00000000000111000000000001100110100000010000000000010100101001000010000100000000000000100000000001010000000000000000000000000000100010000000000100000000000110000000000000000100010100100100100000001000010000000100000000000110000100000100001000001000000001010100000110000010000110000001000100000001001000001010100000000000100011100000011000000000001000100011001100000010100100000000101100000010000000010100000000000100110010000010100000000000001010000000000000001000001000000110001000000101000000000001000000000000000000100000010100010100010000000000000000010100000001000000100010101100000000001111101000000100000000000011001000110001000000010010000000000100001010000000010000010000000001010001000010100000001000000000001000100001000000010000000000001000011000000000010000000000100100000001000001000000000101001100010000011100000010000000010000001000000000100001000000010000011000100011110001000000100000000000000000100001010000001011110010001000010000101000  L-117_2 10111111100111000000001001110111111000011101101000010100111001000010000101011100010010110010100111010011011001001101110110101110101011000100001100110000110110111000001010010110111100100100101011111010010001100100110111110111100110001110101010111010001001010100010110110111010110100111110101011101011100111010110100000111100011100100111001011000001000101111011100001111110110001000111100000110111110010100111011001111111110100010100100011101101010001101001000001000001110000110111110110111101110011001000011010010110000110000011101110101011111000001001100111111010001000000100010101100010000011111101001110101011001110011001110110101110000110111101101101100111110000000111011011011001001110101100110111111101000011111001001100101100100110011111100001110111111111010011101111011100111000111110101110110011111011100110000011100001011110111110011011010001101110011100101011000111000100011111001010111101110001101010011111011011000111111110010011110010001111101  L-202_1 00000000000100100000000000101000010000000010000000000000000010000000000100000000000000100000010011011000001000000001000000000000000011000000000000000000100000001001000010010000100100000100000000000000000000101000000000000110100000000101001001001000010101000000000000000010000010100010100100001100000000110000010100000100100000000000010010100000000000000000000000000000000000000000100000000001000100000100000100000000101000100010000000001000000010000100000101100000001100001010100000000001000000001001000000000000000000000000011101100000000000000000000000010000001100000000100000101100000000000001110100000001001001000000000000100001100000000010000000000101001110000000010000010000000000000000000100000000001010010000000000000000000000110001001000000011000000101010010000100000000100000000000000000000000000001100000000010000000000000000000000101010000000001011000100000000000000000000100000000000100000000000000001101101000100001011010010000100001000011000  L-202_2 10001001001101110000111101101000111000011010011010001000000010011010100110100111001100111000010011011001111001111101100001001000110011000100000000111000110110101001001011111101110100010110101000100011010001101100000000100111100010000111101011101010111111010101000101000111000010100011111100001111110010111001110110001110101011111100010010111100101101100001100000000110000110000001101111010001100100011100111111100001111100110110011111011100010111001100010101100100001111111110100000000001111000001101100111010000001000110000111101100101100011010101000010110111001100010011111100101110001001000111111101000101011101111010101001101001100000100110101101100101101111000100111101011010010101100110110110100000001011011001010011000011100101110101001000000011101101111010010001101010100101010001100010100000001100001100000000010010011000010100101110111111011010101011010101010110011100000011100001000000111010000100110011111111001111001111010010111111001000011001  L-203_1 00000000000000110000000000000000010000000000110000010000001000000101000100010001000000100000000010000001010001000000000000001000000011000000000100000000100110000011001111000000000000000100100000000010000000100100001000000000100100001110101000001000000001010100000000000100000110100010011000001101001100011010000000000001000010100100001001000000000001100000001100000010000110000000000100000001000100000100011110001000111010010000000000000000000010000000000000001000000000000000000000110000100000001001000000000000000000100000000100010000110000000000000000000000001001010000100100100100000000000101000000000111000000011000001000000000100000110010000100000000001100000000110000000000010001110010100110100100001010011001001001000000000000010000000010001010000000000000010001000000100000000001000100100000000100011000010001011100000000010000000100010000000000100000000100000000000000000000110000000000101000000000010001010000010000000100110000001000000000011000  L-203_2 01011010000111110100000001100110010001000010110011010100001011101111001110110101010001110000110011101011010011001011000101001001101011000000010110110000100111101011001111001011000101011110111000011011001000100100101001101111101110111110111100011000011011010101011101100111000110100011111101001101101101111110101000110001101111111101101011101010111101110101101100000010100111011001110100001011100100010100111111011011111010111100110100010111111110001001011101111100000010100110101111110111101000001011110111101110101001110011011111010101110001101011010100110010011001011010101100101100001010000111101001110111001001111001001100100001111101111110001100011110101110011001111001001000111011111010110110101100001110011001011111101101111011111000101110001110001101100011110111111111111111110101100100110111001101111111011001111111011001011101011101111000011000110011111101000000011000000011110001110000101000100100011011111101111001001101110011111111100101111101  L-204_1 10000000000111000000000000100000000000000000000000010010001000001101000100100110000000100010000000000001001001001001000110000001100011000000000000010000100110001110001110000010100100010100100000010010010000000101001001000010100001000010100000000000000001010010001001000100001110100000111101000001000010001010101100001010100010100000001000001000010000100001001101000000000100000010111100011000000110010100011010000100101010110100000000000001100010000100000000001110000010000100000000000101010000000101010100000000000001000000010101010101100100010001000010100100101001000000101000001100011000000100101100001101011001001000000100111001100000010010000100000001001110010000010000011001010000100000100000110000001000000000001001000000100010010000001000001001100000000000010010101000100101000000000000000100000011001110010010001010000000010100000000010010001011110011010000000000001000100110010001000000101000100000000000011100010100111010100000000001000001110010  L-204_2 10001010000111101000001000100010001010110001110001011111011001001101101101100110001010100011000001111011011011001101000110100011100011011010110001011000101110101110001110011110101101010100100000010011010000000101001101110011100101000010101000001100010101010110101001000100001110100010111101110001100011011011111110111010100011111000001010101100111000100101001101010010100100011011111100011001010110110110011110000110101111110110000010001011110110001101001101111111101010100111100011000101110001000101010101000010011101100110010101010111111101010001000110100100111101101000101010101100011000000110101100001101011101011000001100111001100010111010000100100101011110010000011001011001011000100010101110110010001000011010001111111001100111010100101010001011101001010001011011111001110101010100111111100100000111001111010010011010011100010100010010011010001111110111110011010000001110111111110011000100101000101101100010011101011100111111111010011111100101111010  ;  End; |
| **NEX file of 158 SNPs randomly selected from genome** |
| #NEXUS  Begin data;  Dimensions ntax=46 nchar=158;  Format datatype=binary symbols="01" gap=-;  Matrix  H-101_1 10011000000000100000001100100000000000100000100000000000111000010000000100000001000000000000000000100100000001000000000000000000100000000000010001000100000000  H-101_2 10111001101001100100011101100101100010101100110111001001111010111100100100000001010100001010001010111101011011000000000111011011100000001000110001000101010100  H-103_1 00001000001001000000001000100010000000000101000000000001110000000000000000000000010000000010000000101000000010000000000000001000000000000000000000000000000000  H-103_2 11101000101011101100011011111010110110000101001010110001111111101111101101001111011101001111010101111100000111100100000010011000100000010001100001000100100100  H-104_1 00111000000010000100010100100010000000000100000000000001111000010000001000000001000100000000000000110000000010000101000100000000000000000000100000000000000000  H-104_2 00111010000011101100011100100010001100100111001101000011111100111001001000101111110101100001100000111001100111000101001101011000110100100000100111000101101010  H-105_1 00010000000000000000011100001000010010000000000100100001111000000000000000000001010100000010000000100000100010000000000001001000000000100000110000000000000000  H-105_2 00111110001100000111011101101011011110101000100110111001111000101001101010010001010101001111010011110011110011010000001101011011010000101100111001100110110000  H-118_1 01001110000000001100000000001010000000100100000101000100100000010100001010000000010000000010000000000000000000010000000100000000000000000010100000100000000000  H-118_2 11111110100000111100001000101010011011100110100111010110111000011100011110011101110100111111000000100001100011010001001100011011000100011110100001110101110010  H-119_1 10111000000000000000011000100000110000100100000001000101111000010000000000000001000100000111000000100000000001000000000000010000000000000000110000000000010000  H-119_2 10111001001011111100011101111011110000101110000111011101111101011010100101000011010101100111110000100111100011000110000101011100000000000010110101110100110110  H-120_1 00010000000000000000011100000010100100100100000000000000000000001000000000000001000100000000001000000000000010000000000000011000010011000000010001000000100000  H-120_2 10111000101001111100011101101110100100100100100100001000111001011010000110001101000111110001011111110001000011001000100101011000011011010000010001111100111000  H-121_1 00110000100001000100001100100000000000100000000000001001011010001000000000000100000000000000000000011000000011000000000101001000000001000000010011110100000000  H-121_2 10111000101101110101111100101000100100100100100100111111111110011010000100001101011100000101000000111111011011000000001101011010110101000110110011111101010000  H-122_1 00011000000011000000010000000000010000110100000000000101101010000000000000000001000100000000000000100000000010000101000100001000010001000001010001101000000000  H-122_2 10111101101111101100011101101100010011111110010100001111111011011010000111110111100110000111100000101100010010000101100101111010010001111001110001101100100100  H-123_1 00011000000011000000011000001000000000100100000000000101111001011000100000000010000000000000000000100000000010000000000101000000010011000000000001010100000000  H-123_2 10111010101111100100011111101110000101101100000101001101111001011000100000010011010100001101000110111011011011000100100101011010010111000010110101111100011100  H-201_1 00001010100001000000011100101100000000110100010011010100011000000001001000000101010000000100010000100100000010100100000100000100001000010000110001100100110000  H-201_2 10111111100011000110011100101100000000111100111011010101111100011001001100000111110101100111010101100101100110110111001111011100011000010110110001101101110000  L-108_1 00000001000000010000000100100000000000100100000000000100111000001000000000000000000000000000001000100000000010000000000100001000000000000010000001000000101000  L-108_2 10111011101001010101011110110010000001101100100010000101111000111001010000000111000110101001001000111101110011101000010110001000100000100011111111110111111010  L-110_1 00100000000010010000010100000000100000001100000000000101011000001000000000000101000001000000110000100001000011001100000101011000000000000000000001000000010000  L-110_2 10100010011011111101011110011000111010111100101100111101111011111110000110000111010111010101110000111001100111101110101101111011000000100100000001010111111010  L-111_1 00101000000000000000010100100010100000111000000010000000101000000000001100100011000000000001010010100000000010010000000101001000000010010010100001000100110000  L-111_2 00111010001000001000011100100110100101111110001011000001111101011010101100110111010101011101011110100101100111111000001101011010001010011010111001110110110001  L-112_1 00000000000011000000100000100000000000000000000100010101100000000100000100000000010000000000100000101000100000000100000100001010000000000100000000010000000000  L-112_2 10101011110011001110111101101010010111100110100101010101111100011110000100000011010100100011100011111000101111100100010111001110011000000100100001111100000000  L-113_1 00101010000000110100010000100000000000000110000001000100110000011000001000000000000000000010000100100100100010000000000000001000000010000000100001000001000000  L-113_2 10111111011100111110011100101010000110100110000011001100111000111010001000000010011110010111010110101111101011100011011101001100000110000000100001000111100100  L-114_1 00010000000000000000011100101010000000100100000000000100111000000000000000000001110000000100000000100000110110000000000100000000000000000000000001010100000000  L-114_2 11111011000000100000111101101111001011100111001101111111111010111100001111110011110100011101011001111110110110000000000111000000010000011010110001010101100101  L-115_1 00101000010100000000010000101000100000000100000000000101101000001000010100000010000100000010000000100000000010000000000101001000010000000000100001000000000000  L-115_2 10101010110110001100111111101010101100000110100000101101111001011000110110100011010101001011000111100110100011101101001111101101010010100000110001111111000100  L-116_1 00100000000000000000000100100010001100100100000000000000111000011000000000000001000100000000010000001000000000000000000000000000000000000000100001010101000000  L-116_2 00111001100100110100101100111110001100101100010001110101111101011000011000000001000101100111010100111100000110100000010101001001000001010001101001111101100100  L-117_1 00001000101010000000111100101010000000100100000000000001011000000000000100000001010100000000001000100100000010000100000101001000000000000000000001010000000000  L-117_2 00111101101110001000111101111111000000100111010110001101111001001101001110000011010110011111011101101110000011100111011101111001010011110000100011110100010000  L-202_1 00001000000001000000001000000000000001100100010100000000110001001000000000000001000000000000000000100000000000000001000101000000000000000000000001000100000100  L-202_2 10001101100001000110011011101010000001101100010101101100111001001001011110011111000100000111011111111000000011010001000111001000010110010000000101100111010101  L-203_1 00000000000010000000001100000010000100000100000100000101111000001000000010000000000100000000000000100000000010000100000101000000000010000000001001010000000000  L-203_2 11111101111010000011011111101011001101101101010100101101111010011000001110100011010100011101110000110100000110011101101101001000000110000100001001110101011110  L-204_1 00000000000010000000011100000010001000100100000000000101111000001001000000000100001100000010100000010000000010000100000000101000000000000000000001000000000001  L-204_2 10100011001011001100011100101010001100100100100100000101111001001001000011100101011111001011111110110000000010011100001111101000000000011010100001101001110111  ;  End; |

Supplementary Figures

**Supplementary Fig. 1.** The distribution of sequencing depth of the assembled the genome of the Eurasian Tree Sparrow.

**Supplementary Fig.2.** Nucleotide diversity, Tajima’s D and linked disequilibrium values and decay patterns of linkage disequilibrium in the highland and lowland tree sparrows. Orange, highland population; blue, lowland population.

**Supplementary Fig.3.** The genetic structure generated using FRAPPE when K=3, 4 and 5. The colors in each column represent the contribution from each subcluster.

**Supplementary Fig. 4.** Detailed schematics of the five demographic models compared in this study. Arrows represent the presence of gene flow.

**Supplementary Fig.5. A** linear regression to predict the number of capillaries per fiber from the area of oxidative fibers and found the latter statically significantly predicted the former (R^2^=0.6, *F*_1,11_=14.39, *P*<0.01). The plot was generated by “ggplot2.lm” in R and the fit lines were derived from linear regression analysis.

**Supplementary Fig.6.** Genomic landscape and the potential selective regions. Red dots show genomic regions containing genes related to muscle functions.

**

**Supplementary Fig.7.** Gene Ontology (GO) analysis of the genes under the divergent selection revealed 19 significantly perturbed GO terms that are related to muscle processes (38.77%, out of 49 GO terms). Filled bars, *P* value of significance; gray bars, number of genes in each GO.

**Supplementary Fig. 8.** a) GO enrichment in genes found to be differentially expressed in the flight (a) and the cardiac muscle (b) between the highland and lowland tree sparrows, which can be clustered into GO categories related to metabolic and catalytic activity, muscle morphogenesis and contraction, hypoxia response, response to stress, binding and others. Filled bars, *P* value of significance; gray bars, number of genes in each GO.

**Supplementary Fig. 9.** The genes subjected to selection and transcriptional change in the highland tree sparrows are involved in the RhoA/ROCK mediated acto-myosin filament pathway. Genes showing signatures of selection in the highland tree sparrows are in green, and genes showing differentially transcribed are marked in pink. Two inserts show two KEGG pathways (Focal adhesion and Tight junction) where genes under divergent selection (green) and differentially transcribed (pink) were enriched.

**Supplementary Fig. 10.** (a) SNP phylogeny generated from (SNAPP) based on the top divergent SNPs (*F*_ST_>0.24) from 20 candidate selected genes. (b) Principal component analyses of same top divergent SNPs as in panel a. (c) SNP phylogeny generated from (SNAPP) 158 randomly selected SNPs. (d) Number of adaptive alleles (i.e. allelic dosage) at the top divergent SNP positions from 20 muscle related genes (*t*-test, *P*=7.064^e-8^) in highland (orange) and lowland sparrows (blue).

**Supplementary Fig. 11.** Pairwise marginal 2-D SFS plot using in the FASTSIMCOAL analyses.

Supplementary Tables

**Supplementary Table 1.** Summary of DNA libraries and sequencing data of the genome of the Eurasian Tree Sparrow.

| Pair-end libraries | Insert size | Read length | Total Raw data (G) | Raw Sequence coverage (X) | Total Clean Raw data (G) | Clean Sequence coverage (X) |
| --- | --- | --- | --- | --- | --- | --- |
| Illumina reads | 170bp | 90_90 | 75.86 | 69.27 | 70.24 | 64.14 |
|  | 500bp | 90_90 | 57.97 | 50.79 | 53.68 | 47.03 |
|  | 800bp | 90_90 | 17.18 | 14.59 | 15.91 | 13.51 |
|  | 2Kb | 49_49 | 11.96 | 9.29 | 11.07 | 8.60 |
|  | 5Kb | 49_49 | 14.10 | 8.83 | 13.06 | 8.18 |
| Total | |  | 177.07 | 152.77 | 163.96 | 141.46 |

**Supplementary Table 2.** Estimated genome sizes with 17Kmer analysis of the Eurasian Tree Sparrow.

| Species | K-mer number | Peak depth | Genome size (M) | Used Bases (M) | Used Reads | Depth (X) |
| --- | --- | --- | --- | --- | --- | --- |
| Eurasian Tree Sparrows | 45,334,919,774 | 38 | 1,193,024,205 | 55,137,064,590 | 612,634,051 | 46 |

**Supplementary Table 3.** Statistic of the assembly of the genome of the Eurasian Tree Sparrow.

| Species | Scaffold | | Contig | |
| --- | --- | --- | --- | --- |
|  | length(bp) | number | length(bp) | number |
| Total_length | 1,052,976,566 |  | 1,051,087,663 |  |
| Max_len | 39,808,325 |  | 5,063,587 |  |
| number>=100bp |  | 123,682 |  | 128,682 |
| number>=2000bp |  | 2,660 |  | 5,627 |
| N50 | 11,104,764 | 32 | 750,645 | 396 |
| N60 | 7,680,601 | 43 | 583,932 | 554 |
| N70 | 5,151,967 | 59 | 432,097 | 764 |
| N80 | 3,105,589 | 85 | 274,519 | 1,067 |
| N90 | 1,056,811 | 143 | 126,189 | 29,332 |

**Supplementary Table 4**. Proportion of repeats in the genome of the Eurasian Tree Sparrow.

| Type | Repeat Size(bp) | % of genome |
| --- | --- | --- |
| TRF | 41,265,496 | 3.92 |
| RepeatMasker | 64,396,704 | 6.11 |
| RepeatProteinMask | 26,370,483 | 2.50 |
| De Novo | 83,269,436 | 7.90 |
| Total | 124,742,123 | 11.84 |

**Supplementary Table 5**. Comparison of the transponsable elements in the Eurasian Tree Sparrow.

| Type | Length (bp) | % in genome |
| --- | --- | --- |
| DNA | 4,017,770 | 0.38 |
| LINE | 47,164,652 | 4.48 |
| SINE | 86,294 | 0.01 |
| LTR | 29,467,396 | 2.80 |
| Other | 0 | 0.00 |
| Satellite | 2,318,760 | 0.22 |
| Simple_repeat | 1,628,584 | 0.15 |
| Unknown | 6,960,068 | 0.66 |
| Total | 83,269,436 | 7.90 |

**Supplementary Table 6.** Summary of gene models for three types of gene models. P, *ab* initio prediction; H, homology-based; C, cDNA/EST/RNA expressed genes. According to number of gene sources support, the evidence was further divided into single (with one gene source) and more (with two or more gene sources).

| Evidence | >=20% overlap | | >=50% overlap | | >=80% overlap | |
| --- | --- | --- | --- | --- | --- | --- |
|  | Number | % of total | Number | % of total | Number | % of total |
| C(single) | 1 | 0.01 | 32 | 0.19 | 83 | 0.49 |
| C(more) |  | 0 |  | 0 |  | 0 |
| H(single) | 44 | 0.26 | 210 | 1.24 | 656 | 3.88 |
| H(more) | 1,218 | 7.20 | 1,335 | 7.89 | 2,141 | 12.65 |
| P(single) | 8 | 0.05 | 176 | 1.04 | 797 | 4.71 |
| P(more) |  | 0 |  | 0 |  | 0 |
| HC | 304 | 1.80 | 353 | 2.09 | 836 | 4.94 |
| PC | 98 | 0.58 | 101 | 0.60 | 126 | 0.74 |
| PH | 9,674 | 57.16 | 10,147 | 59.95 | 8,267 | 48.84 |
| PHC | 5,578 | 32.96 | 4,279 | 25.28 | 2,712 | 16.02 |

**Supplementary Table 7.** Summary of predicted protein-coding genes and their characteristics. *C. livia (*Rock Pigeon), *C. brachyrhynchos* (American Crow), *F. cherrug* (Saker Falcon), *G. fortis* (Medium Ground Finch), *M. vitellinus* (Golden-collared Manakin*), M. gallopavo* (Wild Turkey), *P. humulis* (Ground Tit), *T. guttata* (Zebra Finch).

| Gene set | | Number | Average gene length (bp) | Average CDS length (bp) | Average exons per gene | Average exon length (bp) | Average intron length (bp) |
| --- | --- | --- | --- | --- | --- | --- | --- |
| *De Novo* | *Augustus* | 19,935 | 16,975 | 1,369 | 8 | 178 | 2,335 |
| Homolog | *C. livia* | 17,141 | 16,186 | 1,329 | 8 | 170 | 2,174 |
|  | *C. brachyrhynchos* | 17,086 | 15,951 | 1,299 | 8 | 168 | 2,173 |
|  | *F. cherrug* | 16,785 | 15,169 | 1,404 | 8 | 179 | 2,010 |
|  | *G. fortis* | 17,616 | 15,828 | 1,301 | 8 | 168 | 2,150 |
|  | *M. vitellinus* | 16,783 | 16,280 | 1,325 | 8 | 168 | 2,179 |
|  | *M. gallopavo1* | 15,191 | 17,181 | 1,488 | 9 | 173 | 2,063 |
|  | *P. humilis* | 18,438 | 16,109 | 1,307 | 8 | 170 | 2,210 |
|  | *T. guttata* | 16,590 | 17,643 | 1,383 | 8 | 168 | 2,253 |
| GLEAN | | 19,048 | 11,998 | 1,278 | 7 | 197 | 1,948 |
| Final set | | 16,925 | 19,175 | 1,560 | 8 | 184 | 2,316 |

**Supplementary Table 8.** Summary of predicted RNA genes and their characteristics. W, whole genome annotation; S, sRNA annotation; I, integrate of whole genome annotation and sRNA annotation.

| Type | | Copy(w) | Average length(bp) | Total length(bp) | % of genome |
| --- | --- | --- | --- | --- | --- |
| miRNA | | 191 | 83.10 | 15,872 | 0.001507 |
| tRNA | | 288 | 74.86 | 21,560 | 0.002048 |
| rRNA | rRNA | 79 | 109.28 | 8,633 | 0.00082 |
|  | 18S | 18 | 65.89 | 1,186 | 0.000113 |
|  | 28S | 56 | 126.39 | 7,078 | 0.000672 |
|  | 5.8S | 0 | 0.00 | 0 | 0 |
|  | 5S | 5 | 73.80 | 369 | 0.000035 |
| snRNA | snRNA | 221 | 117.52 | 25,971 | 0.002466 |
|  | CD-box | 103 | 88.03 | 9,067 | 0.000861 |
|  | HACA-box | 69 | 141.00 | 9,729 | 0.000924 |
|  | Splicing | 34 | 131.71 | 4,478 | 0.000425 |

**Supplementary Table 9**. Functional classification of genes of the Eurasian Tree Sparrow by various databases.

|  | Number | Percent (%) |
| --- | --- | --- |
| Total | 16,925 |  |
| Nr | 16,807 | 99.30 |
| Swissprot | 15,903 | 93.96 |
| KEGG | 14,560 | 86.03 |
| TrEMBL | 16,761 | 99.03 |
| InterPro | 15,855 | 93.68 |
| GO | 11,681 | 69.02 |
| Annotated | 16,833 | 99.46 |
| Unannotated | 92 | 0.54 |

**Supplementary Table 10.** Summarized benchmarks in the BUSCO assessment for the Eurasian Tree Sparrow gene-set

| BUSCO benchmark | Number | Percentage |
| --- | --- | --- |
| Total BUSCO groups searched | 4915 |  |
| Complete single-copy BUSCOs | 4330 | 88.1 |
| Complete duplicated BUSCOs | 54 | 1.1 |
| Fragmented BUSCOs | 311 | 6.3 |
| Missing BUSCOs | 220 | 4.5 |

Note: BUSCO version is: 2.0. The lineage dataset is: aves_odb9 (Creation date: 2016-02-13, number of species: 40, number of BUSCOs: 4915).

**Supplementary Table 11.** Summarized benchmarks in the BUSCO assessment for the Eurasian Tree Sparrow genome assembly.

| BUSCO benchmark | Eurasian Tree Sparrow | |
| --- | --- | --- |
|  | Number | Percentage |
| Total BUSCO groups searched | 4915 |  |
| Complete single-copy BUSCOs | 4627 | 94.1 |
| Complete duplicated BUSCOs | 51 | 1 |
| Fragmented BUSCOs | 145 | 3 |
| Missing BUSCOs | 92 | 1.9 |

Note: BUSCO version is: 2.0. The lineage dataset is: aves_odb9 (Creation date: 2016-02-13, number of species: 40, number of BUSCOs: 4915)

**Supplementary Table 12**. Summary of benchmarks in the genome assessment using the transcripts of the Eurasian Tree Sparrow.

| Dataset | Number | Total length | Bases covered | Sequences covered | with >90% sequence in one scaffold | | with >50% sequence in one scaffold | |
| --- | --- | --- | --- | --- | --- | --- | --- | --- |
|  |  | (bp) | by assembly(%) | by assembly(%) | Number | Percent | Number | Percent |
| >0bp | 60,705 | 52,472,268 | 97 | 97 | 51,809 | 85.35 | 57,533 | 94.77 |
| >200bp | 60,705 | 52,472,268 | 97 | 97 | 51,809 | 85.35 | 57,533 | 94.77 |
| >500bp | 24,922 | 41,580,353 | 98 | 99 | 22,165 | 88.94 | 24,069 | 96.58 |
| >1000bp | 13,812 | 33,831,378 | 98 | 100 | 12,712 | 92.04 | 13,504 | 97.77 |

**Supplementary Table 13.** Sampling information of the Eurasian Tree Sparrow used in population comparative genomic study.

| Group | Sample | Location | Longitude | Latitude | Elevation |
| --- | --- | --- | --- | --- | --- |
| Highland | IOZ14015 | Gangcha, Heimahe | 99.74 | 37.03 | 3213m |
|  | IOZ14018 | Gangcha, Heimahe | 99.74 | 37.03 | 3213m |
|  | IOZ14019 | Gangcha, Heimahe, | 99.74 | 37.03 | 3213m |
|  | IOZ14020 | Gangcha, Heimahe | 99.74 | 37.03 | 3213m |
|  | IOZ6498 | Qinghai lake, Heimahe | 99.47 | 36.59 | 3200m |
|  | IOZ6499 | Qinghai lake, Heimahe | 99.47 | 36.59 | 3200m |
|  | IOZ6500 | Qinghai lake, Heimahe | 99.47 | 36.59 | 3200m |
|  | IOZ6501 | Qinghai lake, Heimahe | 99.47 | 36.59 | 3200m |
|  | IOZ6502 | Qinghai lake, Heimahe | 99.47 | 36.59 | 3200m |
|  | IOZ6503 | Qinghai lake, Heimahe | 99.47 | 36.59 | 3200m |
|  |  |  |  |  |  |
| Lowland | IOZ4735 | Qinhuangdao | 119.35 | 39.56 | 100m |
|  | IOZ4736 | Qinhuangdao | 119.35 | 39.56 | 100m |
|  | IOZ4737 | Qinhuangdao | 119.35 | 39.56 | 100m |
|  | IOZ4738 | Qinhuangdao | 119.35 | 39.56 | 100m |
|  | IOZ4739 | Qinhuangdao | 119.35 | 39.56 | 100m |
|  | IOZ4750 | Qinhuangdao | 119.35 | 39.56 | 100m |
|  | IOZ14001 | Bejing | 116.35 | 39.93 | 59m |
|  | IOZ14002 | Bejing | 116.35 | 39.93 | 59m |
|  | IOZ14003 | Bejing | 116.35 | 39.93 | 59m |
|  | IOZ14004 | Bejing | 116.35 | 39.93 | 59m |
|  | IOZ18809 | Tianjin | 117.10 | 39.10 | 70m |

**Supplementary Table S14.** Statistics of reads mapping and coverage of 11 highland and 12 lowland Eurasian Tree Sparrow individuals used in population genomics.

| Group | Sample | ID | Clean reads (G) | Coverage | Mapping rate (%) |
| --- | --- | --- | --- | --- | --- |
| Highland | IOZ14015 | H-101 | 17.94 | 15.40 | 98.80 |
|  | IOZ14018 | H-103 | 15.52 | 13.15 | 98.55 |
|  | IOZ14019 | H-104 | 18.13 | 15.34 | 98.75 |
|  | IOZ14020 | H-105 | 18.24 | 15.66 | 98.77 |
|  | IOZ6498 | H-118 | 16.45 | 14.00 | 98.80 |
|  | IOZ6499 | H-119 | 14.19 | 12.07 | 98.79 |
|  | IOZ6500 | H-120 | 14.72 | 12.50 | 98.86 |
|  | IOZ6501 | H-121 | 16.85 | 14.27 | 98.66 |
|  | IOZ6502 | H-122 | 18.89 | 16.03 | 98.75 |
|  | IOZ6503 | H-123 | 23.13 | 19.51 | 98.86 |
|  |  | H-201 | 16.42 | 13.8 | 97.96 |
| Lowland | IOZ4735 | L-202 | 21.79 | 18.59 | 98.79 |
|  | IOZ4736 | L-203 | 20.83 | 17.81 | 98.93 |
|  | IOZ4737 | L-108 | 19.86 | 16.75 | 98.86 |
|  | IOZ4738 | L-204 | 22.32 | 19.01 | 94.66 |
|  | IOZ4739 | L-110 | 20.13 | 17.08 | 98.90 |
|  | IOZ4740 | L-111 | 28.55 | 24.24 | 98.76 |
|  | IOZ14001 | L-112 | 19.15 | 16.16 | 98.77 |
|  | IOZ14002 | L-113 | 20.32 | 17.27 | 98.80 |
|  | IOZ14003 | L-114 | 20.75 | 17.74 | 98.73 |
|  | IOZ14004 | L-115 | 28.88 | 24.31 | 98.76 |
|  | IOZ14006 | L-116 | 19.71 | 16.65 | 98.67 |
|  | IOZ18809 | L-117 | 33.53 | 28.44 | 98.72 |

**Supplementary Table 15.** Population demographic parameters between highland and lowland populations.

| Populations | Nucleotide diversity | Tajima’s D |
| --- | --- | --- |
| Highland | 0.00227 (95%CI:9.70^e-6^-0.004) | 1.17 (95%CI:0-2.079) |
| Lowland | 0.00226 (95%CI:9.78^e-6^-0.004) | 1.24 (95%CI:0-2.17) |

**Supplementary Table 16.** CV error values for Structure analysis (k=1-5).

| Structure | CV error |
| --- | --- |
| K=1 | 0.63893 |
| K=2 | 0.84007 |
| K=3 | 1.21449 |
| K=4 | 1.50206 |
| K=5 | 1.82203 |

**Supplementary Table 17. Model selection using FASTSIMCOAL and AIC.**

|  | Models | MaxEstLhood | N of estimated params | Ln-Lhood | AIC | ∆AIC | AIC’s weight 或(W) |
| --- | --- | --- | --- | --- | --- | --- | --- |
| M1 | One-single population with constant size | -7574078.57 | 5 | -34879920.81 | 34879930.81 | 12425 | 0 |
| M2 | One-single population with changing size | -7785532.06 | 5 | -35853700.12 | 35853710.12 | 986205 | 0 |
| M3 | Colonization with unidirectional gene flow from highland to lowland | -75771379.46 | 7 | -34867490.95 | 34867504.95 | 0 | 1 |
| M4 | Colonization with unidirectional gene flow from lowland to highland | -7574125.71 | 7 | -34880137.89 | 34880151.89 | 12647 | 0 |
| M5 | Colonization with bi-directional gene flow between lowland and highland | -7572153.12 | 9 | -34871053.8 | 34871071.8 | 3566 | 0 |
| M6 | Colonization with unidirectional gene flow from lowland to highland and population size changing | -7574808.22 | 9 | -34883280.97 | 34883298.97 | 15794 |  |
| M7 | Colonization with unidirectional gene flow from highland to lowland and population size changing | -7574407.41 | 9 | -34881435.19 | 34881453.19 | 13948 |  |
| M8 | Colonization with bi-directional gene flow and population size changing | -7574226.93 | 12 | -34880604.01 | 34880628.01 | 13123 |  |

**Supplementary Table 18.** Parameter estimates for the best-fit demographic model (divergence with gene flow model)*.* All estimates assume diploid cells, a one-year generation time, and a nuclear mutation rate of 3.3E-9 per site per generation (Zhang et al. 2014). Point estimates are provided with 95% confidence intervals in parentheses. Point estimates are those identified in the best-fit run of the 20 model selection replicates. Abbreviations include: *N*_e_ = effective population size, T_DIV_ = timing of divergence, *m* = migration probabilities (or, the probability that any gene from one population transfers to another on a per generation basis).

| Parameter | Description | Estimate |
| --- | --- | --- |
| *Effective population sizes* | |  |
| N_highland | Highland *N*_e_ | 85,058 (44,294-185,163) |
| N_lowland | Lowland *N*_e_ | 94,612 (49,204-206,121) |
| N_ANC | Ancestral *N*_e_ | 279,539 (221,836-293,081) |
| *Colonization times* | |  |
| TDIV | Split leading to colonization to the Qinghai-Tibet Plateau | 2,598 (1,179-4,567) |
| *Migration probabilities* | |  |
| MIG12 | *m* from lowland into highland | 0.97e^-6^ (0.93e^-6^-1.20e^-6^) |
| N2M12 | Individuals per generation from lowland into highland | 0.08 (0.04-0.22) |

**Supplementary Table 19.** Fiber area, perimeter and capillary number of the flight muscle of the highland and lowland tree sparrows. Data are presented as means and standard error (SE). Sample and group means were compared using LGM in SPSS. Differences were taken as significant for *P*<0.05.

| Muscle phenotype | N | Highland tree tree sparrows | Lowland tree sparrows | P value |
| --- | --- | --- | --- | --- |
| Fiber area | 12 | 759.03±16.23 | 581.5±21.02 | *F* _1, 11_ =44.7, *P*<0.001 |
| Fiber perimeter | 12 | 101.93±1.43 | 88.05±1.42 | *F* _1, 11_ =47.29, *P*<0.001 |
| Capillary number per fiber | 12 | 1.93±0.049 | 1.632±0.041 | *F* _1, 11_ =21.41, *P*<0.001 |

**Supplementary Table 20.** Myocyte diameter and capillary number of the cardiac muscle of highland and lowland tree sparrows. Data are presented as means and standard error (SE). Sample and group means were compared using LGM in SPSS. Differences were taken as significant for *P*<0.05.

| Muscle phenotype | N | Highland tree sparrows | Lowland tree sparrows | P value |
| --- | --- | --- | --- | --- |
| Myocyte diameter | 14 | 5.114±0.074 | 4.954±0.142 | *F* _1, 13_ =2.99, *P*=0.1 |
| Capillary number | 14 | 438416±170.15 | 3304.33±26.17 | *F* _1, 13_ =30.84, *P*<0.001 |

**Supplementary Table 21.** The muscle genes showed signal of positive selection in the highland tree sparrows.

| Genomic region | Gene ID | Gene name | *F*_ST_ | *D*_XY_ | Nucleotide diversity (θπ) | Tajima’s *D* | LD | Function |
| --- | --- | --- | --- | --- | --- | --- | --- | --- |
| scaffold171:595452-632052 | PMO015697 | DCHS1 | 0.103 | 0.0019 | 0.0019 | 2.04 | 0.11 | Heart development |
| scaffold26:11278718-11293669 | PMO014180 | IGFBP3 | 0.114 | 0.0005 | 0.0004 | 1.691 | 0.348 | Heart development |
| scaffold136:94488-120095 | PM004556 | ARHGAP39 | 0.117 | 0.0004 | 0.0004 | 1.852 | 0.363 | Rho GTPase-activating proteins |
| scaffold41:182968-297118 | PMO007732 | PREX1 | 0.044 | 0.0007 | 0.0007 | 1.639 | 0.387 | Guanine nucleotide exchange factor for Rac |
| scaffold96:1393771-1467190 | PMO004351 | CTNNA3 | 0.025 | 0.0008 | 0.0008 | 2.172 | 0.170 | Muscle system process |
| scaffold96:1629011-1790073 | PMO004349 | CTNNA3 | 0.092 | 0.0008 | 0.0007 | 3.77 | 0.308 | Muscle system process |
| scaffold2:5288764-5373485 | OMO005973 | MLLT4 | 0.021 | 0.0012 | 0.0012 | 1.569 | 0.141 | Tight junction |
| scaffold96:1309377-1336150 | PMO004352 | TRIO | 0.026 | 0.0009 | 0.0009 | 2.112 | 0.157 | Rho guanine nucleotide exchange factors (GEFs) |
| scaffold1:24700001-24850000 | PMO011578 | ARHGAP15 | 0.027 | 0.0019 | 0.0019 | 1.93 | 0.055 | Rho GTPase-activating proteins |
| scaffold2:24982871-25019461 | PMO006054 | ARHGAP18 | 0.031 | 0.0023 | 0.0023 | 1.832 | 0.080 | Rho GTPase-activating proteins |
| scaffold103:1822253-1953450 | PMO004700 | COL11A1 | 0.064 | 0.0036 | 0.0036 | 2.213 | 0.069 | Muscle development |
| scaffold190:329325-387116 | PMO005060 | APC | 0.023 | 0.0011 | 0.0011 | 1.882 | 0.143 | Cytoskeleton proteins |
| scaffold20:13303403-13511317 | PMO012958 | ITPR2 | 0.056 | 0.0003 | 0.0003 | 3.287 | 0.467 | Vascular smooth muscle contraction |
| scaffold149:314056-374189 | PMO005042 | MYO7A, | 0.062 | 0.001 | 0.001 | 1.647 | 0.118 | Myosin protein |
| scaffold99:1659832-2111042 | PMO010563 | NBEA | 0.018 | 0.0029 | 0.0029 | 1.978 | 0.063 | Cytoskeleton |
| scaffold1:5835372-5914613 | PMO011608 | OBSCN | 0.03 | 0.0025 | 0.0024 | 2.311 | 0.065 | Rho guanine nucleotide exchange factors (GEFs) |
| scaffold18:11220338-11310103 | PMO006640 | SPTBN5 | 0.038 | 0.0031 | 0.003 | 2.158 | 0.066 | Cytoskeleton |
| scaffold19:10675769-11013073 | PMO002522 | PTPPRM | 0.016 | 0.0027 | 0.0027 | 2.062 | 0.065 | Cell adhesion molecules |
| Scaffold4:12509267-12612135 | PMO007663 | FAT3 | 0.027 | 0.0024 | 0.0023 | 2.037 | 0.068 | Cadherins |
| Scaffold79:374025-447289 | PMO010315 | TBC1D32 | 0.042 | 0.004 | 0.004 | 2.161 | 0.099 | Heart development |

**Supplementary Table 22.** Pathways enriched for positively selected genes detected by SweepFinder analysis. Bold highlighted the pathways that were detected in *F*_ST_ and θπ analyses

| Geneset | Enriched pathways | Input number | FDR |
| --- | --- | --- | --- |
| Top 1% (154 genes) | Calcium signaling pathways | 6/161 | 0.074094 |
|  | **Adherens junction** | **4/70** | **0.074221** |
|  | **Tight junctions** | **5/122** | **0.080839** |
|  | Wnt signaling pathways | 5/126 | 0.084722 |
|  |  |  |  |
| Top 2% (248 genes) | **Adherens junction** | **4/70** | **0.076062** |
|  | Calcium signaling pathways | 6/161 | 0.079231 |
|  | **Tight junctions** | **5/122** | **0.091558** |
|  | Gap junction | 4/81 | 0.093496 |
|  | Wnt signaling pathway | 5/126 | 0.093496 |
|  |  |  |  |
| Top 5% (458 genes) | Calcium signaling pathway | 12/161 | 5.8e-05 |
|  | Oocyte meiosis | 8/92 | 0.0086 |
|  | **Neuroactive ligand-receptor interaction** | 14/267 | 0.0099 |
|  | Notch signaling pathway | 5/44 | 0.022 |
|  | Wnt signaling pathway |  | 0.0285 |
|  | **Focal adhesion** | **10/187** | **0.0289** |
|  | **Adherens junction** | **5/70** | **0.0656** |
|  | Adrenergic signaling in cardiomyocytes | 6/123 | 0.11 |
|  | Vascular smooth muscle contraction | 5/103 | 0.144 |
|  | **Tight junction** | **5/122** | **0.2** |

**Supplementary Table 23.** Statistic of transcriptomic sequencing data.

| Tissue | Group | Clean reads | Mapped reads | Percent mapped to genome (%) |
| --- | --- | --- | --- | --- |
| Flight muscle |  |  |  |  |
| Low_07 | Lowland | 17,166,225 | 15,504,534 | 90.32% |
| Low_09 | Lowland | 20,441,592 | 18,002,910 | 88.07% |
| Low_10 | Lowland | 16,679,631 | 14,457,904 | 86.68% |
| Low_11 | Lowland | 20,923,512 | 17,881,233 | 85.46% |
| High_02 | Highland | 16,690,586 | 14,589,241 | 87.41% |
| High_08 | Highland | 17,278,802 | 15,141,414 | 87.63% |
| High_10 | Highland | 16,849,769 | 14,802,522 | 87.85% |
| High_11 | Highland | 17,144,248 | 14,857,205 | 86.66% |
| Cardiac muscle |  |  |  |  |
| Low_14 | Lowland | 21,447,101 | 19,109,367 | 89.10% |
| Low_19 | Lowland | 17,223,560 | 15,299,688 | 88.83% |
| Low_09 | Lowland | 20,244,087 | 17,636,649 | 87.12% |
| High_06 | Highland | 17,350,254 | 15,256,078 | 87.93% |
| High_07 | Highland | 17,116,185 | 15,087,917 | 88.15% |
| High_08 | Highland | 17,288,719 | 15,504,523 | 89.68% |

**Supplementary Table 24.** Description for differential expressed genes in the cardiac muscle between highland and lowland tree sparrows.

**See attached Excel file.**

**Supplementary Table 25.** Description for differential expressed genes in flight muscle between highland and lowland tree sparrows.

**See attached Excel file.**

**Supplementary Table 26.** Top 5% enriched pathways in the genes under the divergence selection and differentially transcribed.

| Top 5% pathways in genes under divergent selection | Top 5% pathways in genes under the differentially transcribed in cardiac muscle | Top 5% genes under the differentially transcribed in flight muscle | Shared pathways between gene-sets |
| --- | --- | --- | --- |
| Adherens junctions (Kegg04520) | ECM-receptor interaction (Kegg04512) | Lysosome (Kegg04142) | Tight junction (Kegg04530) |
| Glutathione metabolism (Kegg04514)  Tight junction (Kegg04530) | Focal adhesion (Kegg04510) | Focal adhesion (Kegg04510) | Focal adhesion (Kegg04510) |
|  | Proteoglycans in cancer (Kegg05205) | Purine metabolism (Kegg00230) |  |
|  | Phagosome (Kegg04145) | Nicotinate and nicotinamide metabolism (Kegg 00760) |  |
|  | Amoebiasis (Kegg05146) | ECM-receptor interaction (Kegg04512) |  |
|  | Complement and coagulation (Kegg04610) | Tight junction (Kegg 04530) |  |
|  |  | Regulation of actin cytoskeleton (Kegg04810) |  |
|  |  |  |  |

**Supplementary Table 27.** The number of high elevation adaptive alleles across all the tree sparrows individuals (926 adaptive alleles in 87 genes and 152 adaptive alleles in 20 muscle genes).

| Populations | Individuals | 926 adaptive alleles in 87 genes | 152 adaptive alleles in 20 muscle genes |
| --- | --- | --- | --- |
| Highland | H-101 | 1835 | 200 |
|  | H-103 | 1584 | 204 |
|  | H-104 | 1945 | 201 |
|  | H-105 | 1805 | 243 |
|  | H-118 | 1868 | 212 |
|  | H-119 | 1859 | 210 |
|  | H-120 | 1890 | 206 |
|  | H-121 | 1905 | 218 |
|  | H-122 | 1939 | 235 |
|  | H-123 | 1924 | 224 |
|  | H-201 | 1971 | 229 |
| Lowland | L-108 | 1143 | 113 |
|  | L-110 | 1054 | 119 |
|  | L-111 | 1121 | 122 |
|  | L-112 | 1191 | 170 |
|  | L-113 | 1177 | 140 |
|  | L-114 | 1189 | 171 |
|  | L-115 | 1079 | 118 |
|  | L-116 | 1019 | 124 |
|  | L-117 | 1233 | 177 |
|  | L-202 | 1124 | 173 |
|  | L-203 | 1161 | 143 |
|  | L-204 | 1177 | 112 |
|  |  |  |  |

**Supplementary Table 28.** Sampling information used in histological analyses and RNA-seq of the flight muscle.

| Group | Sample | location | Histological work | RNA-seq |
| --- | --- | --- | --- | --- |
| Highland population | High_01 | Heimahe | √ |  |
|  | High_02 | Heimahe | √ | √ |
|  | High_08 | Heimahe | √ | √ |
|  | High_10 | Heimahe | √ | √ |
|  | High_11 | Heimahe | √ | √ |
|  | High_12 | Heimahe | √ |  |
| Lowland population | Low_07 | Beijing | √ | √ |
|  | Low_09 | Beijing | √ | √ |
|  | Low_10 | Beijing | √ |  |
|  | Low_11 | Beijing | √ |  |
|  | Low_15 | Beijing | √ | √ |
|  | Low_20 | Beijing | √ | √ |
| Hypoxia group | Exp_01 | Beijing | √ |  |
|  | Exp_02 | Beijing | √ |  |
|  | Exp_03 | Beijing | √ |  |
|  | Exp_04 | Beijing | √ |  |
|  | Exp_05 | Beijing |  |  |
| Control group | Con_01 | Beijing | √ |  |
|  | Con_02 | Beijing | √ |  |
|  | Con_04 | Beijing | √ |  |
|  | Con_04 | Beijing | √ |  |
|  | Con_05 | Beijing |  |  |

**Supplementary Table 29.** Sampling information used in histological analyses and RNA-seq of the cardiac muscle.

| Group | Sample | location | Histological work | RNA-seq |
| --- | --- | --- | --- | --- |
| Highland population | High_01 | Heimahe | √ |  |
|  | High_02 | Heimahe | √ |  |
|  | High_03 | Heimahe | √ |  |
|  | High_05 | Heimahe | √ |  |
|  | High_06 | Heimahe | √ | √ |
|  | High_07 | Heimahe | √ | √ |
|  | High_08 | Heimahe | √ | √ |
| Lowland population | Low_07 | Beijing | √ |  |
|  | Low_08 | Beijing | √ |  |
|  | Low_14 | Beijing | √ | √ |
|  | Low_15 | Beijing | √ |  |
|  | Low_16 | Beijing | √ |  |
|  | Low_18 | Beijing | √ |  |
|  | Low_19 | Beijing | √ | √ |
|  | Low_09 | Beijing | √ | √ |
| Hypoxia group | EXP_01 | Beijing | √ |  |
|  | EXP_02 | Beijing |  |  |
|  | EXP_03 | Beijing | √ |  |
|  | EXP_04 | Beijing | √ |  |
|  | EXP_05 | Beijing | √ |  |
| Control group | Con_01 | Beijing | √ |  |
|  | Con_02 | Beijing | √ |  |
|  | Con_03 | Beijing | √ |  |
|  | Con_04 | Beijing | √ |  |
|  | Con_05 | Beijing |  |  |

**Supplementary Table 30.** Likelihood distributions and goodness of fit tests (*t*-tests) of the two separate analyses for FASTSIMCOAL models.

| Models | Model definitions | Analyses | Likelihood values | *t*-tests of two analyses |
| --- | --- | --- | --- | --- |
| M1 | Single population with constant size | Run 1 | -7576017 - -7574081 | P=0.1 |
|  |  | Run 2 | -7575879 - 7574079 |  |
| M2 | Single population with changing size | Run 1 | -7799660 - -7785532 | P=0.63 |
|  |  | Run 2 | -7799974 - -7788882 |  |
| M3 | Highland colonization with unidirectional gene flow form lowland to highland | Run 1 | -7585396 - -7571435 | P=0.57 |
|  |  | Run 2 | -7583989 - -7571379 |  |
| M4 | Highland colonization with unidirectional gene flow form highland to lowland | Run 1 | -7582411 - -7574126 | P=0.73 |
|  |  | Run 2 | -7582411 - -7574159 |  |
| M5 | Highland colonization with bidirectional gene flow between highland and lowland | Run 1 | -7580808 - -7572220 | P=0.13 |
|  |  | Run 2 | -7610017 - -7572153 |  |
| M6 | Highland colonization with unidirectional gene flow form lowland to highland and population size changing | Run 1 | -7582742 - -7574860 | P=0.99 |
|  |  | Run 2 | -7582918 - -7574808 |  |
| M7 | Highland colonization with unidirectional gene flow form highland to lowland and population size changing | Run 1 | -7578968 - -7574460 | P=0.92 |
|  |  | Run 2 | -7581662 - -7574407 |  |
| M8 | Highland colonization with bidirectional gene flow and population size changing | Run 1 | -7589540 - -7574326 | P=0.40 |
|  |  | Run 2 | -7594077 - -7574227 |  |

**Supplementary Table 31.** Myocyte diameter and capillary number of the highland and lowland tree sparrows, represented for left and right ventricles, respectively. Data are presented as means and standard error (SE). Sample and group means were compared using pair t-test in SPSS.

| Muscle phenotypes |  | Highland tree sparrow | |  |  | Lowland tree sparrows | |  |
| --- | --- | --- | --- | --- | --- | --- | --- | --- |
|  | N | Left ventricle | Right ventricle | P value | N | Left ventricle | Right ventricle | P value |
| Myocyte diameter | 7 | 5.27±0.13 | 5.18± 0.11 | P=0.82 | 7 | 4.95± 0.16 | 4.98±0.10 | P=0.57 |
| Capillary number | 4 | 4529±222 | 4418±6.36 | P=0.60 | 4 | 3316±65.74 | 3251±126.54 | P=0.614 |
